# Identification of Conserved ABC Importers Necessary for Intracellular Survival of *Legionella pneumophila* in Multiple Hosts

**DOI:** 10.3389/fcimb.2017.00485

**Published:** 2017-11-30

**Authors:** Amrita Lama, Samuel L. Drennan, Rudd C. Johnson, Grace L. Rubenstein, Eric D. Cambronne

**Affiliations:** Department of Molecular Microbiology and Immunology, Oregon Health and Science University, Portland, OR, United States

**Keywords:** *Legionella*, pathogenesis, transposon mutagenesis, *Acanthamoeba castellanii*, macrophage, Dot/Icm T4b secretion system, ABC transporter, *Francisella*

## Abstract

It is established that the human pathogen *Legionella pneumophila* becomes significantly augmented for infection of macrophages after intracellular growth in amoebae when compared to like-strains cultivated in laboratory media. Based on this observation, we reasoned that the most critical virulence determinants of *L.p*. are expressed by responding to stimuli generated by the protozoan host specifically; a process we term “protozoan-priming.” We sought to identify *L.p*. virulence factors that were required for replication in amoebae in order to highlight the genes necessary for production of the most infectious form of the bacterium. Using a transposon mutagenesis screen, we successfully identified 12 insertions that produced bacteria severely attenuated for growth in amoebae, while retaining a functional Dot/Icm type IVb secretion system. Seven of these insertion mutants were found dispensable for growth in macrophages, revealing attractive therapeutic targets that reside upstream of the pathogen-human interface. Two candidates identified, *lpg0730* and *lpg0122* were required for survival and replication in amoebae and macrophage host cells. Both genes are conserved among numerous important human pathogenic bacteria that can persist or replicate in amoebae. Each gene encodes a component of an ATP binding cassette (ABC) transport complex of unknown function. We demonstrate the *lpg0730* ortholog in *Francisella tularensis* subsp. *novicida* to be essential for colonization of both protozoan and mammalian host cells, highlighting conserved survival mechanisms employed by bacteria that utilize protozoa as an environmental reservoir for replication.

## Introduction

*Legionella pneumophila* (*L.p*.) is a Gram-negative bacterium predominantly associated with freshwater environments. Free-living bacteria persist in water, yet replication is restricted to the confines of host protozoan cells (amoebae), where the bacterium is a facultative intracellular parasite (Fields, 1996; Abu Kwaik et al., 1998). It is an opportunistic human pathogen, where egress from host amoebae generates the most infectious form of the bacterium (Cirillo et al., 1994, 1999; Brieland et al., 1996). Aerosolization of contaminated water sources provides an invariant route of transmission, providing access to resident lung alveolar macrophages. Intracellular colonization and replication in host macrophages occurs in a similar fashion to the bacterial life-cycle in protozoa, manifesting a spectrum of pathologies collectively termed “legionellosis.” The range of presentation, which is largely owed to host immune-competence, includes self-limited flu-like illness (Pontiac fever) to a severe and often fatal pneumonia called Legionnaires' disease (Burillo et al., 2017).

In order to survive and replicate in eukaryotic cells, *L.p*. requires a specialized type IVb secretion system termed Dot/Icm (Marra et al., 1992; Berger and Isberg, 1993; Segal and Shuman, 1997). This multi-protein secretion apparatus functions to deliver up to 300 effector proteins into the host-cell (Zhu et al., 2011). Effectors collectively function in the subversion of host-cellular processes to facilitate establishment of a replication-permissive compartment, often termed *Legionella*-containing vacuole (LCV) (Roy, 2002). Strains defective for Dot/Icm-mediated transport of effectors are rendered largely avirulent (Roy et al., 1998). *L.p*. developed these intracellular survival strategies through close associations with protozoa in the environment. Strategies adopted for survival in protozoa therefore directly translated to efficient replication in the hostile environment encountered within the macrophage of the lung.

**Figure 1:**
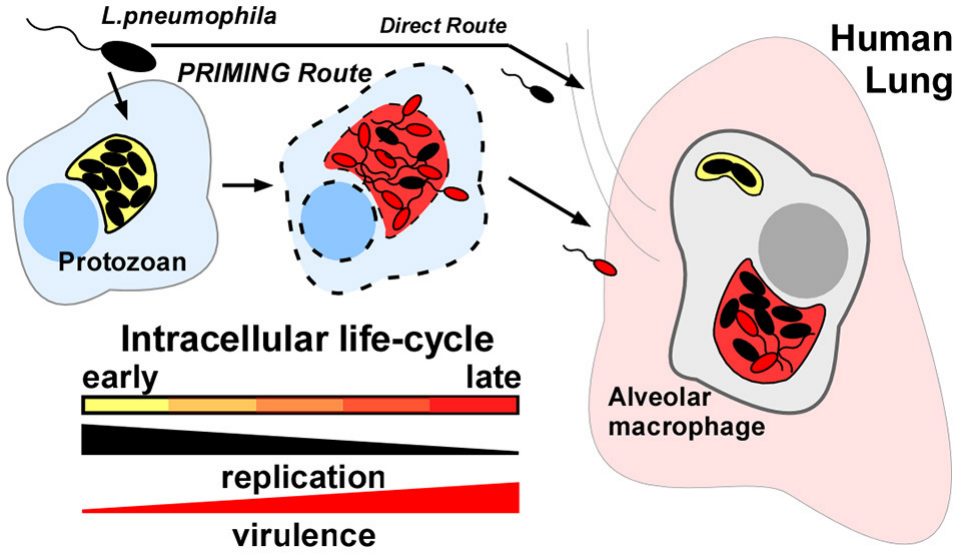
Protozoan-primed *Legionella pneumophila* are augmented for dissemination to human macrophages. *L.p*. strains that have completed an intracellular life-cycle in natural-host protozoa are up to 100x more infectious (*Priming route*) than like-strains cultivated in laboratory medium or persisting in freshwater (*Direct route*). Response to specific host-cellular cues must trigger expression of key virulence determinants for alveolar macrophage infection in the human lung.

Importantly, evidence dating back over 20 years demonstrated that *L.p*. cultivated in amoebae prior to infection of macrophage cell lines or murine hosts were both hyper-invasive and hyper-virulent; upwards of 100-fold when compared to like-strains cultured in laboratory media; a phenomenon we term “protozoan-priming” (Figure 1) (Cirillo et al., 1994, 1999; Brieland et al., 1996, 1997a,b; Drennan et al., 2013). This effect has been experimentally ascribed to a transition from an intracellular replicative phase to a non-dividing transmissive form (Molofsky and Swanson, 2004). Similarly “transmissive” bacteria have been modeled *in vitro* by culturing *L.p*. to early stationary phase. Based on these early observations however, the discrepancy between the virulence properties associated with protozoan-primed vs. *in vitro* cultured bacteria could be exploited to reveal key determinants for *L.p*. dissemination.

We speculated that *L.p*. must respond to environmental stimuli provided exclusively by the protozoan host-cell in order to activate the expression of genes that contribute to the observed augmented infection phenotypes. With this notion in mind, we first sought to comprehensively identify genes necessary for intracellular survival in amoebae specifically, to potentially uncover therapeutic targets that would prevent generation of the highly virulent and most biologically relevant form of *L.p*. in the context of human infection. Additionally, these targets would have potential to reside upstream of the bacterial-human interface.

To this end, we conducted a genome-wide transposon mutagenesis screen in *L.p*. using intracellular survival and replication in the model protozoan *Acanthamoeba castellanii* (*A.c*.) as a primary evaluation criterion (Holden et al., 1984). Mutants that were attenuated or failed to replicate in *A.c*. (as determined using fluorescence microscopy) were next subjected to additional rounds of screening. Potential *dot*/*icm* insertions were selected-against based on sensitivity to growth on artificial media containing sodium chloride, and confirmed for a functional Dot/Icm transporter using an adenylate-cyclase reporter assay (Vogel et al., 1996; Cambronne and Roy, 2007). Host-cell specificity among several candidate insertions was demonstrated by examining intracellular replication in additional host cell types including murine and human macrophages.

Of particular interest were insertions in *lpg0730* and *lpg0122*, each encoding a structural component of a distinct ATP binding cassette (ABC) transport complex (Theodoulou and Kerr, 2015). The assignment of substrate to each transport complex is unresolved. However, we found both *loci* conserved among several bacterial pathogens that can utilize protozoa as an intermediate reservoir for proliferation and transmission in the environment. Further, we demonstrated that disruption of the *lpg0730* ortholog in *Francisella tularensis* subsp. *novicida* was essential for colonization of both protozoan and mammalian host-cells. Our data suggest that Lpg0730-containing ABC transport complexes therefore represent a conserved intracellular survival determinant that represents an attractive target for inhibiting proliferation in environmental host cells.

## Results

### Construction and screening of *L. pneumophila* mutant library

**Figure 2:**
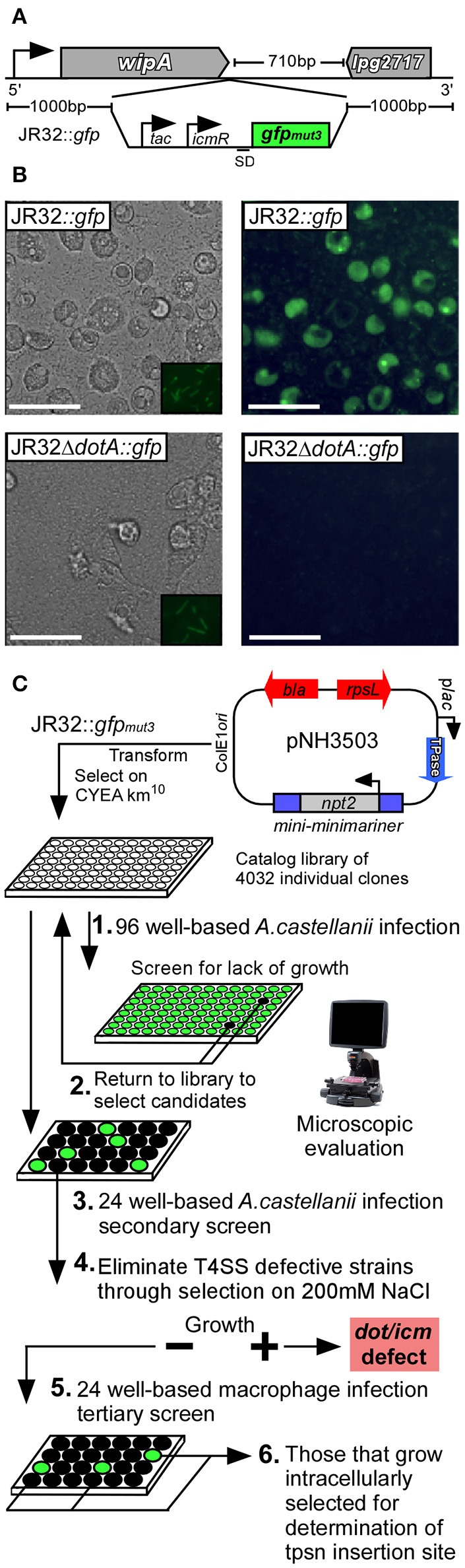
Schematic representation of genome-wide transposon mutagenesis of *L. pneumophila* and survival screen in multiple host-cell types. **(A)** *gfp*_*mut*3_ with tandem promoters (*tac, icmR*) was inserted on the *L.p*. JR32 chromosome 3′ to the *wipA* (*lpg2718*) locus using homologous recombination. **(B)** The JR32::*gfp* strain was used to generate a T4SS defective mutant through in-frame deletion of *dotA*, resulting in JR32Δ*dotA*::*gfp*. Both strains were cultured to post-exponential phase in the presence of IPTG (insets) and used to infect *A.c*. cells. Eighteen hours post-infection, light (left column) and fluorescence (right column) micrographs were captured to visualize replication-permissive vacuoles. Scale bar = 50 μm. **(C)** JR32::*gfp* was subjected to transposon mutagenesis using the plasmid pNH3503, where kanamycin resistant clones were transferred to 96-well culture plates. Each clone was cultured to post-exponential phase and used to infect *A.c*. cultures for 18 h (1). Individual wells were examined microscopically to evaluate abundance of fluorescent vacuoles. Mutants that lacked fluorescence were targeted for secondary infections of *A.c*. using controlled MOI (2–3). Mutants that passed secondary screen were subjected to cultivation on media supplemented with 150 mM NaCl in order to select against Dot/Icm-defective strains (4). Salt-sensitive mutants were subsequently used to infect CHO FcγRII-transgenic, J774.A1, and THP-1 monolayers to evaluate survival capacity using fluorescence microscopy (5). All remaining mutants were subjected to DNA sequencing to determine transposon insertion sites (6).

We first generated a fluorescently-tractable isogenic *L.p*. strain harboring a single copy of *gfp*_mut3_ on the chromosome that would serve as a wild-type representation for mutagenesis (JR32*::gfp*) (Cormack et al., 1996). GFP production was driven by dual promoters in tandem. The isopropyl β-D-1-thiogalactopyranoside (IPTG)-inducible *tac* promoter was located immediately 5′ to the *icmR* promoter; active in early stationary phase (Neild and Roy, 2003). The *gfp* construct was inserted 3′ to the stop codon of the *wipA* effector locus (Figure 2A). This location was chosen due to the large 710 bp stretch of non-coding sequence 3′ to the monocistronic *wipA*. WipA, which was recently reported to harbor tyrosine phosphatase activity had been previously determined dispensable for intracellular survival of *L.p*. in both macrophage and amoebae (Ninio et al., 2005; Pinotsis and Waksman, 2017). We additionally constructed an isogenic Δ*dotA::gfp* strain for use as a negative control for intracellular replication (Roy et al., 1998). Both JR32*::gfp* and JR32Δ*dotA::gfp* produced GFP when cultured *in vitro* in the presence of IPTG (Figure 2B/*insets*). However, WT was the only strain capable of supporting intracellular replication, which could be detected using fluorescence microscopy (Figure 2B).

In order to provide unbiased coverage of the non-essential *L.p*. genome, we constructed a library of individual insertion mutants of *L.p*. strain JR32*::gfp* using a modified minimariner transposon (mini-minimariner) mutagenic strategy (Murata et al., 2006). For library construction, we used pNH3503 plasmid carrying the mini-minimariner transposon. This transposon targets TA di-nucleotides on the chromosome, integrating in a random fashion (Figure 2C) (Murata et al., 2006). Greater than 4,000 insertions were isolated using 20 individual rounds of mutagenesis, where 200–250 isolates were collected per round. PCR analysis of randomly selected mutants demonstrated the presence of transposon in every isolate examined (not shown). Individual mutants were cataloged in 96-well format for preservation (Figure 2C).

To screen the library, we first examined whether each mutant could replicate intracellularly in the model host protozoan *A.c*. (Holden et al., 1984). Individual mutants were used to infect *A.c*. in 96-well plate format for 18 h. Infected wells were examined via fluorescence microscopy. Mutants that were attenuated or unable to replicate intracellularly, as judged by reduction or absence of mature fluorescent vacuoles, were selected for further rounds of screening (Figure 2C).

A secondary screen of candidate mutants was performed in 24-well format under controlled multiplicity of infection. Successful candidates that were validated as attenuated or defective in intracellular replication were next tested for their capacity to grow under conditions of elevated sodium chloride concentration. WT *L.p*. cannot grow on media containing (150–200 mM) NaCl, a consequence of a functional Dot/Icm type IVb secretion system (Vogel et al., 1996). Conversely, *L.p*. mutations in several *loci* encoding structural components of the Dot/Icm transporter were shown to render *L.p*. permissive for growth on elevated [NaCl] (Figure 2C). This important selection criterion therefore allowed for identification of isolates that failed to divide in host cells yet presumably retained a functional Dot/Icm transporter. Candidates that satisfied the screening criteria were next used to infect a panel of mammalian host-cell types to evaluate specificity. Thirty-eight insertions were selected for further characterization, where 18 failed to replicate in *A.c*. and an additional 20 were attenuated by 50% or greater when compared to WT (as measured subjectively by total mature replication vacuoles per microscopic field).

### Sequence validation of transposon insertion sites

Of the 38 isolates, 24 were successfully sequence validated, where transposon insertion sites were determined either by using the transposon insertion as a site for priming and subsequent amplification of purified genomic DNA or with “arbitrary” PCR (O'Toole and Kolter, 1998).

Twelve of the isolates were found in *dot/icm*-encoding *loci*, where insertions were limited to eight of the 27 genes that comprise the transport system (Table 1). These eight *dot/icm* genes therefore represented a functionally distinct class, as each failed to grow on elevated [NaCl]. An additional 12 insertions were located at the sites described in (Table 2). Each of these mutants were severely attenuated or failed to replicate in one of the four eukaryotic hosts examined. The remaining 14 mutants, displaying a range of intermediate phenotypes in *A.c*. infection were not sequenced but were cataloged. Infection phenotypes for these isolates are described in (Table S1).

**Table 1:** NaCl-sensitive *dot/icm* insertions.

**Isolate**	**Insertion**	**Annotation**
B4-3	*lpg2674*	*dotD*
A3-4	*lpg0453*	*dotE/icmC*
E9-4	*lpg0454*	*dotP/icmD*
E2-9	*lpg0446*	*dotL/icmO*
E2-16	*lpg0446*	*dotL/icmO*
H12-32	*lpg0446*	*dotL/icmO*
C11-13	*lpg0445*	*dotM/icmP*
G9-32	*lpg0445*	*dotM/icmP*
F5-40	*lpg0445*	*dotM/icmP*
B7-29	*lpg0445*	*dotM/icmP*
E7-27	*lpg0455*	*dotN/icmJ*
A2-28	*lpg0444*	*icmQ*

*Exhibited zero growth in A.c., CHO-FcγRII, J774.A1, THP-1*.

**Table 2:** Sequence-validated gene insertions identified in screen.

**Isolate**	**Insertion**	**Annotation**	**Function**	**Intracellular survival (% of WT infection)**			
				***A.c*.^a^**	**CHO^b^**	**J774**	**THP-1**
**C4-1**	***lpg0730***	***perM***	**Membrane permease**	**0**	**0**	**100**^c^	**0**^c^
H2-16	*lpg0730*	*perM*	Membrane permease	0	0	100	0
C9-34	*lpg0730*	*perM*	Membrane permease	0	0	100	0
C6-28	*lpg2276*		Glu/Leu/Phe/Val dehydrogenase	0	0	100	100
A9-13	*lpg1811*	*lysC*	Aspartokinase-diaminopimelate decarboxylase	0	100	100	100
H2-15	lpg2422-23	lem25-*hyp*	Dot/Icm effector Lem25 - hypothetical	0	0	50	50
**D1-37**	***lpg0122***		**ABC transporter-ATP binding protein**	**10**	**10**	**100**	**10**^c^
C1-1	*lpg0376*	***sdhA***	Dot/Icm effector protein SdhA	10	10	10	0
G12-15	*lpg0133*	*proQm*	Activator of ProP osmoprotectant transporter	10	50	50	100
D12-34	*lpg1362-63*	*gspG-gspF*	Type II secretion	10	100	100	0
A12-18	*lpg1369*	*htpG*	Heat shock protein Hsp90	50	100	100	100
A9-20	*lpg1796-97*	*lysR*-rvfA	LysR family transcriptional regulator – Dot/Icm effector protein RvfA	50	100	100	100

*Red indicates genes targeted in this study. Blue indicates Dot/Icm effector-encoding genes*.

*If two genes are indicated for an isolate, insertion was in intergenic region*.

a *Acanthamoeba castellanii*.

b *Opsonized bacteria used to infect CHO FcγRII monolayers*.

c *As measured over 72 h using CFU counts*.

In addition to measuring survival and replication in *A.c*., each mutant was used to infect FcγRII transgenic Chinese Hamster Ovarian (CHO) cells (Nagai et al., 2005). Here bacteria were opsonized with polyclonal antisera directed against heat-killed WT *L.p*. prior to infection, which effectively stimulated phagocytosis and allowed for measure of survival in an artificial host system. Mutants were also used to infect the murine macrophage cell line J774.A1, or phorbol myristate acetate (PMA)-differentiated human THP-1 macrophages. Survival values indicated in Table 2 represent approximates of the total number of fluorescent vacuoles per microscopic field compared as a percentage to the total number of observed vacuoles in an infection using WT *L.p*. over the same time-course of infection.

Overall, the 24 mutants identified using the selection criteria could be divided into two classes: (I) *dot/icm* machinery mutants that retained salt-sensitivity, or (II) mutants that were attenuated for intracellular survival in one or multiple host cell types. We found some degree of host cell-specificity associated with survival among seven of the 12 class 2 insertions (Table 2). Additionally, 3 of 12 class II insertions were located in intergenic regions.

Of the insertions identified as a result of limited or complete failure to produce mature replication vacuoles in *A.c*., three interrupted *dot/icm* effector encoding genes. The product of *sdhA* has been implicated in vacuolar integrity and is necessary for survival in macrophages (Creasey and Isberg, 2012). The promoter regions of effector-encoding *lem25* (*lpg2422*) and *rvfA* (*lpg1797*) were also interrupted (Huang et al., 2011). Complementation studies using plasmid-borne copies of *lpg2422* or *lpg2423* (encoded opposite direction from transposon insertion) in the H2-15 isolate failed to restore intracellular survival. Further the *lpg1797* ORF alone, or in the context of native promoter failed to complement the A9-20 isolate. Therefore, the contribution of these transposon insertions to observed phenotypes remains unresolved.

Two additional insertions were found to interrupt genes encoding enzymes involved in amino acid metabolism. *lpg2276* encodes a Glu/Leu/Phe/Val- family dehydrogenase, a NAD^+^ or NADP^+^-dependent enzyme that de-aminates the amino acid to a keto-acid form, which can be assimilated into the Kreb's cycle. The *lpg1811* locus encodes aspartokinase-diaminopimelate decarboxylase, an enzyme important in lysine synthesis. The requirement for this gene was strictly limited to intracellular survival in the protozoan host.

Additional sequenced insertions included the *proQm* activator of ProP osmoprotectant transporter, which functions on ProP at a post-translational level. ProP responds to osmotic stress functioning as a zwitterion/proton symporter (Chaulk et al., 2011). Loss of *proQm* was found more detrimental for survival in the protozoan host. The D12-34 insertion was located between *lspG* and *lspF*, structural components of the type II secretion system in *L.p*. The Lsp secretion system has been previously implicated in intracellular survival of *L.p*. through secretion of multiple enzymes to the extracellular confines of the replication-permissive vacuolar compartment (Cianciotto, 2005). We also found *hsp90* to be important for optimal survival in the protozoan cell, remaining dispensable in each of the metazoan cell types.

### Identification of putative ABC import complexes

**Figure 3:**
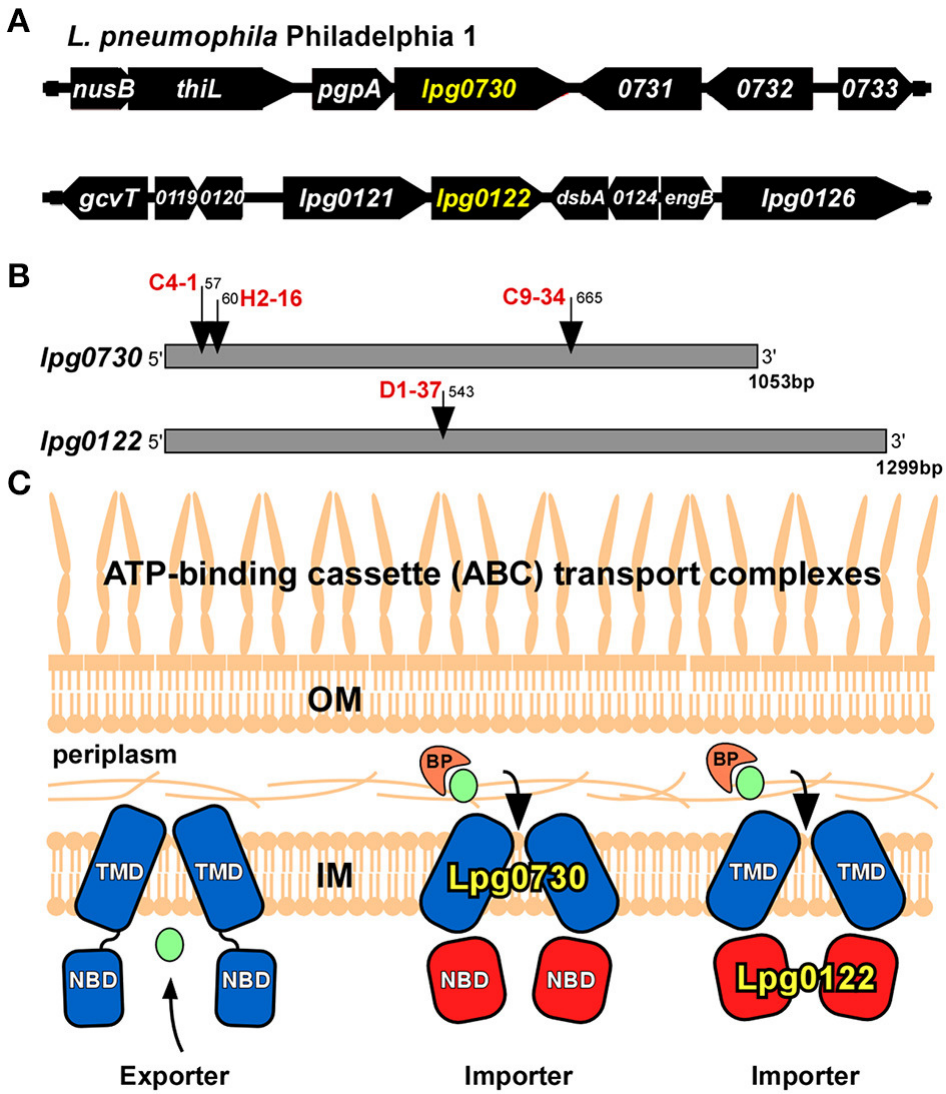
Transposon insertions found in ABC import complex components**. (A)** Location on the *L.p*. genome of *lpg0730* and *lpg0122*. **(B)** Transposon insertion sites (nucleotide relative to +1) on the *lpg0730* and *lpg0122* ORF's for candidates identified in screen with clone designations in red. **(C)** Proposed location and function of Lpg0730 and Lpg0122 in the *L.p*. inner membrane. BP, binding protein (orange); TMD, transmembrane domain (blue); NBD, nucleotide binding domain (red); IM, inner membrane; OM, outer membrane, transported substrate depicted in green.

Of particular interest, was that four of the insertions interrupted putative ATP-binding cassette (ABC) transporter components, *lpg0730* and *lpg0122*. (Table 2) (Theodoulou and Kerr, 2015). The *lpg0730* locus is the final gene in a transcriptional unit that contains as many as 9 ORFs. It resides 3′ to the gene encoding phosphatidylglycerophosphate phosphatase (*pgpA*) involved in lipid metabolism and membrane homeostasis (Figure 3A) (Funk et al., 1992). Three unique transposon insertion sites were identified in the screen, highlighting the importance of this locus for survival in *A.c*. (Figure 3B). The 1,053 bp *lpg0730* ORF encodes a 350 aa (38.7 kDa) protein with 9 putative transmembrane segments. It is annotated as a membrane permease of the UPF0118 superfamily, which encompasses many substrate-binding transporters. *E. coli* YdiK is the archetype of this family of proteins, whose function is not determined but it is a member of the *purR* regulon, responsive to purine concentration (Cho et al., 2011). These permeases share strong homology with a class of transmembrane domain (TMD) proteins found in ABC transport systems.

The *lpg0122* locus is the second gene in a bi-cistronic transcriptional unit (Figure 3A). The D1-37 isolate was interrupted by the transposon at nucleotide position 543 of the 1,299 bp *lpg0122* ORF (Figure 3B). The gene encodes a 432 aa (48.4 kDa) protein annotated as a nucleotide binding domain (NBD) component of an ABC transport system. Its sequence falls under the NtrD/SsuB transporter family that encompasses numerous ATP-binding cassette domains that are involved in the import of nitrates and sulfonates. Lpg0122 shares its N-terminal 252 aa with TauB, involved in taurine import (Pereira et al., 2015). The C-terminal region is annotated as an AAA-ATPase associated region. The product of *lpg0121* is annotated as an ABC membrane permease (TMD).

We surmised *lpg0730* and *lpg0122* individually represented components of distinct ABC “*importer”* complexes, which could be extrapolated based on amino acid sequence and their arrangement on the *L.p*. genome. For ABC import complexes, the TMD and NBD components are generally distinct proteins, whereas in ABC exporters the TMD and NBD are each sub-domains of a single fusion protein (Figure 3C) (Theodoulou and Kerr, 2015). It is more likely that Lpg0122 (NBD) associates with the co-expressed Lpg0121 (TMD) than with Lpg0730. Furthermore, transposon interruptions of *lpg0730* or *lpg0122* exhibited different requirements for intracellular survival based on host cell type. Both Lpg0730 and Lpg0122 were determined to be highly conserved in Gram-negative and some Gram-positive bacteria when translated protein was used as template for homology search. This included several notable human pathogens that can use freshwater amoebae as an environmental intermediate. Multiple sequence alignment and phylogenetic assignment of Lpg0730, Lpg0122, and corresponding orthologous proteins are depicted in Figures S1, S2, respectively.

Intracellular survival relative to WT was grossly estimated visually for *lpg0730::Tn* and *lpg0122::Tn* as part of the screening process. In addition to *A.c*., where survival rates approximated zero and 10 percent respectively, polyclonal anti-*legionella*-opsonized strains were used to infect FcγRII-transgenic CHO monolayers to measure survival independent from internalization (Nagai et al., 2005). Here, both insertions generated identical survival phenotypes to those observed in *A.c*. When used to infect murine J774.A1 macrophages, *lpg0730* and *lpg0122* were found dispensable for intracellular survival, and performed identical to WT. These results suggested that each of these *loci* were critical for survival in particular host cell types (*A.c*., CHO FcγRII) while remaining completely dispensable in another (J774A.1). Curiously, although *lpg0122::Tn* also performed similar to WT during infection of human THP-1 macrophages, each of the three *lpg0730::Tn* mutants failed to replicate, suggesting a gradient of substrate concentration or availability among the macrophage hosts (Table 2).

### Host cell-specific activation of candidate genes

**Figure 4:**
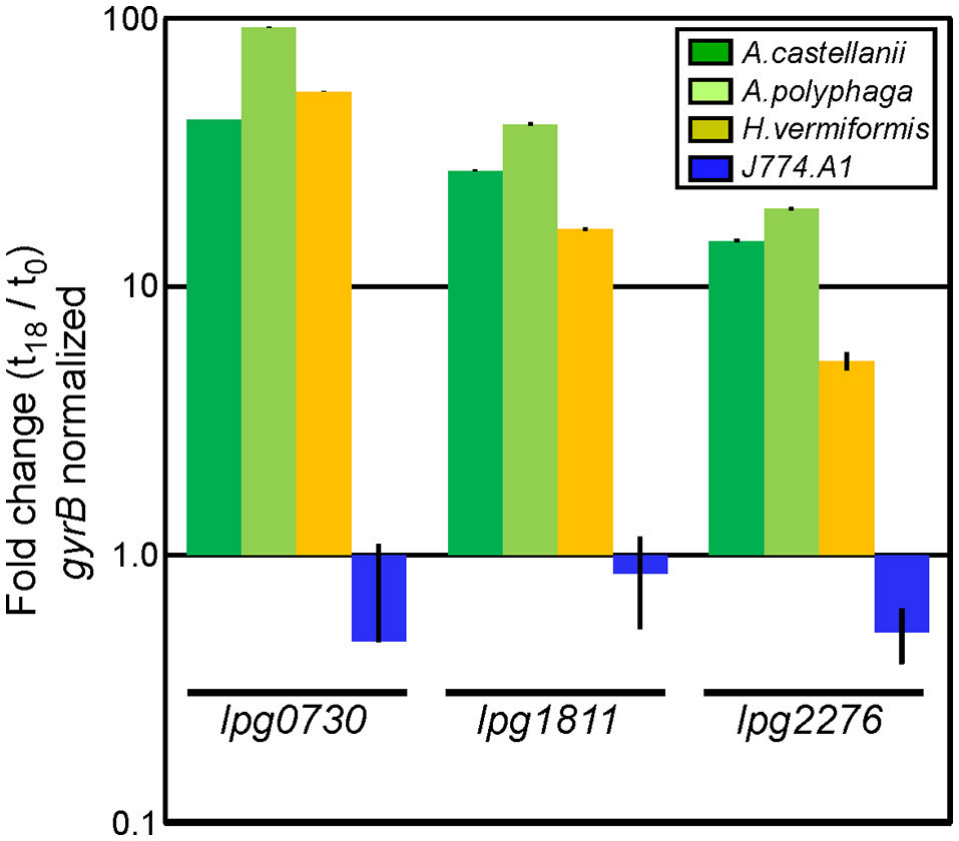
Genes identified in screen are exclusively activated during infection in protozoa. qPCR analysis using total bacterial RNA isolated immediately after exposure to, or 18 h post-infection of indicated host cells. Bars indicate fold-change of indicated transcripts (t_18_/t_0_) normalized to DNA gyrase B (*gyrB*). Infections were performed in triplicate (three biological replicates) with standard deviations indicated.

Some degree of host cell-specificity was observed for seven of 12 of the non-*dot/icm* insertion mutants. Differential genetic requirements for intracellular survival were most pronounced in *lpg0730, lpg1811*, and *lpg2276* insertion mutants (Table 2). We sought to determine the expression profile of these *loci* in WT *L.p*. in the context of intracellular growth in multiple established host cell types. Two closely related *Acanthamoeba* species (*A. castellanii, A. polyphaga*), a more distantly related amoebae species (*Hartmanella vermiformis*), and J774.A1 macrophages were selected for the analyses. Total RNA was isolated from bacteria either immediately after exposure to host cells or 18 h post-infection (prior to host cell egress). Quantitative PCR was performed using primer sets to generate ~150 bp amplicons from *lpg0730, lpg1811, lpg2276*, and DNA gyrase B (*gyrB*), which was used for normalization. As depicted in Figure 4 each of the genes examined were highly activated in all three amoebae species while remaining inactivated or slightly repressed in J774.A1 over the time-course of infection. These results suggest that the micro-environments encountered by *L.p*. in various host phagosomes must be chemically diverse.

### Characterization of *lpg0730* and *lpg0122* mutant strains

**Figure 5:**
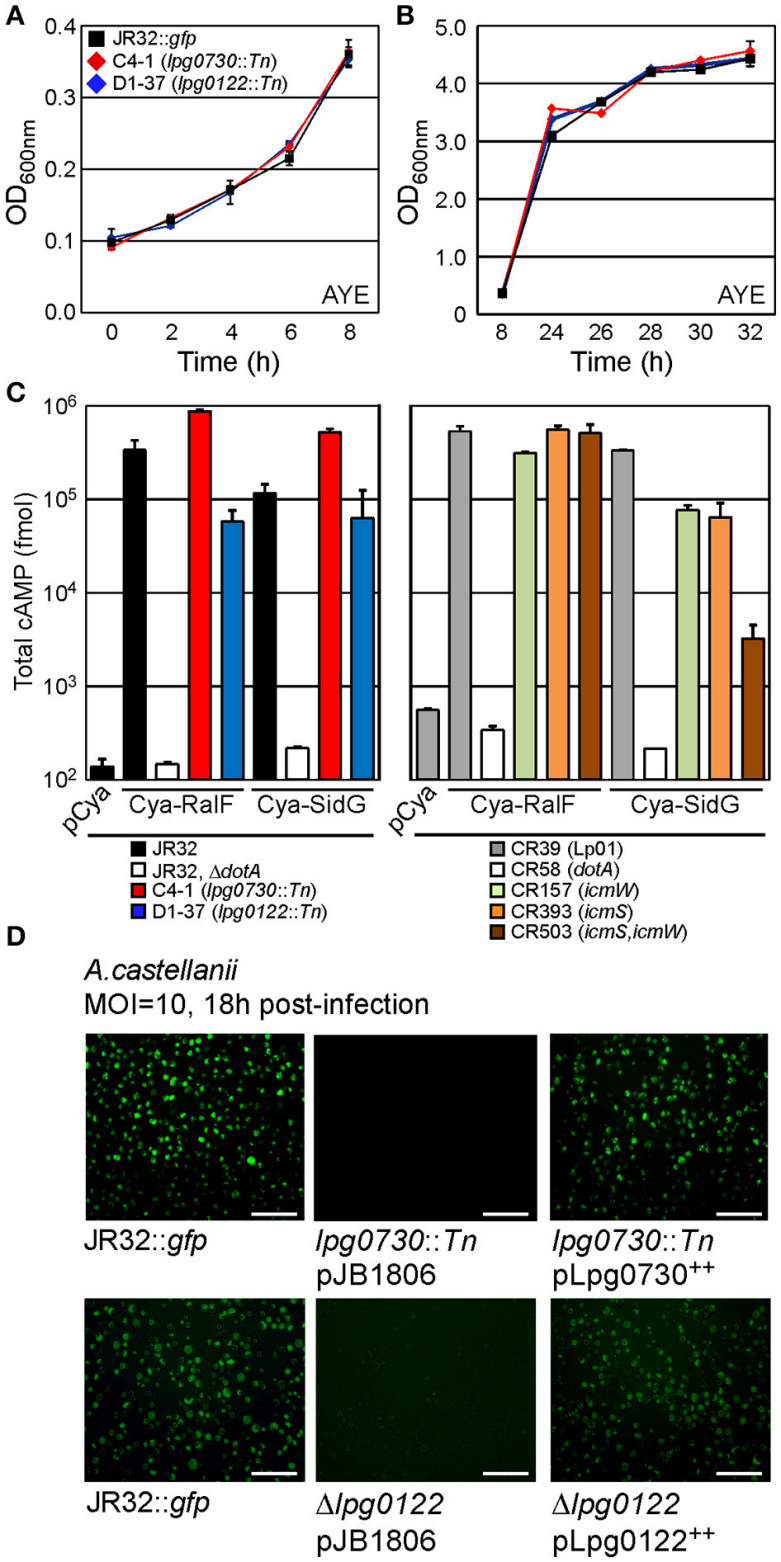
*lpg0730* and *lpg0122* are dispensable for replication *in vitro* and do not interfere with Dot/Icm-mediated translocation of effectors. **(A)** Indicated strains were suspended in AYE broth at an OD_600 nm_ = 0.1 and cultivated at 37°C on shaker. Spectrophotometric measurements were taken at indicated times spanning lag to exponential phase transition. **(B)** As in **(A)** with measurements taken to cover late exponential to early stationary phase transition. **(C)** *L.p*. strains (indicated with colored bars) harboring plasmids producing adenylate cyclase (Cya) fusions to the *L.p*. effectors RalF or SidG were used to infect CHO FcγRII monolayers for 1 h. Cells were lysed after which total cAMP was extracted and quantified using immunosorbent assay. Bars indicate total cAMP produced per well (fmol). Experiments were performed in triplicate (three biological replicates) with standard deviations indicated. **(D)** *lpg0730*::*Tn* and Δ*lpg0122* were transformed with either empty vector (pJB1806) or plasmids that supported inducible expression of Lpg0730 or Lpg0122. Resulting strains were cultured to post-exponential phase in parallel with JR32::*gfp* and used to infect *A.c*. cultures for 18 h (MOI = 10). Representative fluorescence micrographs are shown for infected *A.c*. cells with each indicated bacterial strain. Fluorescent *foci* represent individual replication vacuoles. Scale bar = 100 μm.

Our mutagenesis strategy selected only for non-essential genes. We therefore examined growth kinetics of the C4-1 (*lpg0730*) and D1-37 (*lpg0122*) insertion mutants through direct comparison to WT cultured in ACES-buffered yeast extract broth (AYE). As shown in Figures 5A,B the growth kinetics of all three strains were indistinguishable. Similar results were obtained through culture in defined synthetic media (not shown) (Warren and Miller, 1979). These results indicate that both *lpg0730* and *lpg0122* are dispensable for *L. p*. growth *in vitro*.

It is possible that the severe intracellular growth attenuation observed for the C4-1 and D1-37 insertion mutants in *A.c*. was a result of detrimental effects on the type IV secretion pathway, which is essential for pathogenesis. We sought to determine whether interruptions in *lpg0730* or *lpg0122* generated strains that were defective for *dot/icm*-dependent translocation of effector proteins. Reporter proteins were utilized that consisted of the catalytic domain of the calmodulin-dependent adenylate cyclase (Cya) from *Bordetella pertussis* fused to the amino terminus of the established effectors RalF or SidG (Cambronne and Roy, 2007). The production of cyclic adenosine-monophosphate (cAMP) resulting from the translocation of a Cya-effector hybrid into CHO-FcγRII cells was used to measure productive translocation. As indicated in Figure 5C, both the Cya-RalF and Cya-SidG hybrids were translocated to the host cytosol with higher fidelity in the C4-1 (*lpg0730*) background than in the parent JR32 strain. Translocation of Cya-RalF in the D1-37 (*lpg0122*) strain was slightly attenuated when compared with JR32. However, there was no significant difference in translocation efficiency of Cya-SidG, an *icmSW*-dependent effector protein. Cya-SidG translocation was previously reported to be attenuated with deletions in either *icmS* or *icmW*, which form an adaptor complex in the *L.p*. cytoplasm that promotes delivery of a class of effectors to the substrate receptor complex (Figure 5C) (Cambronne and Roy, 2007). Even though SidG translocation is reduced in *icmS* or *icmW* mutants, both strains can form replication-permissive vacuoles with reduced kinetics in *A.c*. (Coers et al., 2000; Tilney et al., 2001). It is likely that a mutation in *lpg0122* does not have a direct effect on type IV secretion, but may indirectly affect kinetics of vacuole maturation.

Both *lpg0730* and *lpg0122* were next targeted for deletion using homologous recombination in a tri-parental mating scheme. We successfully generated a marker-less deletion of the *lpg0122* locus (Δ*lpg0122*) on the JR32::*gfp* genetic background. Multiple attempts to generate a similar deletion of the *lpg0730* locus, including production of variant recombination vectors, failed to generate the desired strain. We therefore continued our studies using the C4-1 transposon insertion.

We next performed genetic complementation *in trans* by cloning *lpg0730* or *lpg0122* into a low-copy plasmid, with expression guided by an IPTG-inducible *tac* promoter. Sequence validated plasmids were transformed either into the C4-1 (*lpg0730*) or Δ*lpg0122* strains, in parallel to empty vector (pJB1806). Parent JR32, C4-1, and Δ*lpg0122* harboring appropriate plasmids were cultured to early stationary phase and used to infect *A.c*. After 18 h of infection *A.c*. cultures were imaged using light and fluorescence microscopy. A productive infection with WT JR32::*gfp* can be visualized in Figure 5D. No vacuoles were visualized with the C4-1 (*lpg0730*) strain, and the *lpg0122* deletion phenocopied the D1-37 isolate, with sparse vacuoles and low fluorescence. Production of Lpg0730 or Lpg0122 *in trans* fully restored *A.c*. infection fidelity to WT levels when evaluated microscopically (Figure 5D).

**Figure 6:**
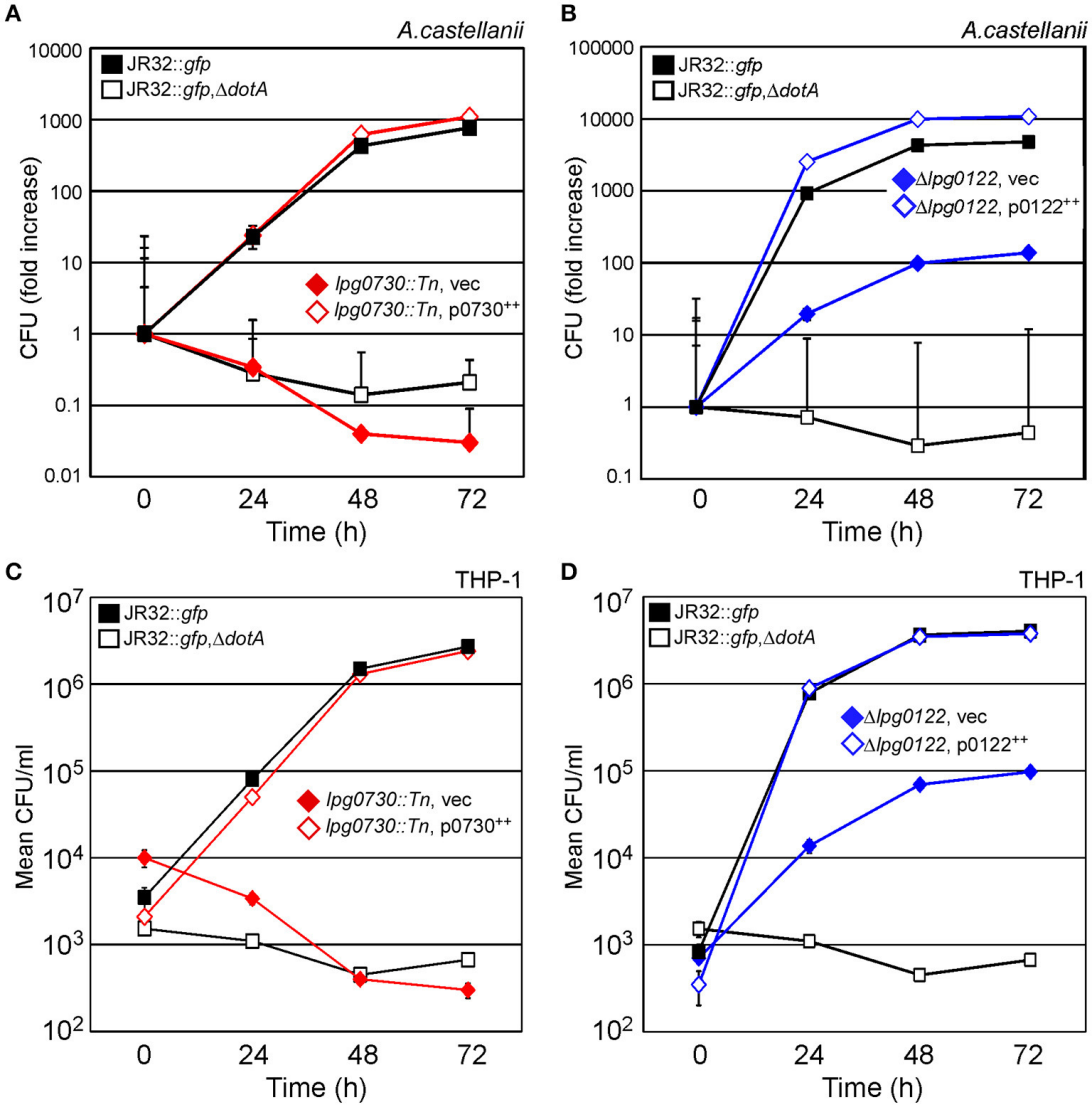
Intracellular survival and replication of *lpg0730::Tn* and Δ*lpg0122* in *A.c*. and THP-1 human macrophages is fully restored via complementation *in trans*. **(A,B)** Indicated strains were used to infect *A.c*. cultures (MOI = 1), where host cells were lysed at indicated times and total CFU were determined by colony count after serial dilution and cultivation on CYEA. Results are shown as fold increase over 72 h. Experiments were performed in triplicate (three biological replicates) with standard deviations indicated. **(C,D)** As in **(A,B)** with 48 h PMA-differentiated THP-1 monocytes substituted for *A.c*. Graphs indicate mean CFU/ml (three biological replicates) with standard deviations indicated.

To quantitatively evaluate contribution of *lpg0730* and *lpg0122* to intracellular survival of *L.p., lpg0730::Tn* (C4-1) or Δ*lpg0122* were used to infect either *A.c*. or THP-1 macrophages over a 72 h time course. Parallel infections were performed with WT (JR32::*gfp*), *dotA* deletion (JR32::*gfp*, Δ*dotA*), or complement strains. The *lpg0730*::*Tn* strain failed to replicate in either *A.c*. (Figure 6A) or THP-1 (Figure 6C), and was less persistent than Δ*dotA* in both hosts. Production of Lpg0730 via low-copy plasmid restored survival and replication of *lpg0730*::*Tn* to WT levels in *A.c*. and THP-1 (Figures 6A,C). The *lpg0122* deletion strain (Δ*lpg0122*) was replication competent in both *A.c*. and THP-1 with significantly reduced kinetics. In *A.c*., maximal CFU counts were achieved 72 h post-infection and were nearly 100-fold lower than CFU's recovered in the WT infection (Figure 6B). Similar attenuation was observed over the infection time course in THP-1 (Figure 6D). Similar to Lpg0730, plasmid-derived Lpg0122 restored intracellular survival and replication to WT levels in both *A.c*. and THP-1 (Figures 6B,**D**). Overall both expression constructs complemented the growth attenuation observed in the two mutant strains.

### Conservation of *lpg*0730 and *lpg*0122 in pathogens that colonize protozoa

In addition to *Legionella* species, many notable human pathogens are capable of colonizing protozoan cells. *Mycobacterium, Salmonella, Staphylococcus*, and many other Gram-negative and Gram-positive species can use amoebae as a nutrient rich environmental intermediate for replication and dissemination. We used the amino acid sequences of Lpg0730 and Lpg0122 to search for homologous and orthologous proteins in a subset of pathogenic species known to colonize amoebae use the BLASTP algorithm. To our surprise, both proteins were conserved in nearly every bacterial species examined. We next performed multiple sequence alignment and phylogenetic analysis using Clustal Omega. Of the species examined, Lpg0730, Lpg0122, and their respective homologs/orthologs separated into three clades from a common ancestral protein. The phylogenetic arrangement and multiple sequence alignments for Lpg0730 and Lpg0122 are shown in Figures S1, S2 respectively.

We sought to determine whether Lpg0730 homologs/orthologs were necessary for intracellular survival. We first constructed a C-terminal FLAG epitope-tagged version of Lpg0730 by cloning this construct into the same vector used for complementation. Using the same *A.c*. infection strategy employed in Figure 5D, WT (JR32::*gfp*), and *dotA* deletion (JR32::*gfp*, Δ*dotA*), and *lpg0730::Tn* (C4-1) pLpg0730_*FLAG*_ were evaluated microscopically 18 h post-infection. Similar to expression of Lpg0730 alone appending the C-terminal FLAG epitope had no detrimental effect on intracellular survival and the fidelity of infection was restored to WT levels (Figure S3).

Lpg0730 is assigned by sequence to the PerM family of membrane permeases, which is exemplified by the YdiK protein in *E. coli*. Alignment of Lpg0730 and YdiK is shown in Figure S1. We sought to determine whether an Lpg0730 homolog from *L. longbeachae* (76% identity) or PerM orthologs from *Salmonella enterica* subsp. Typhimurium (25.2% identity) or *Francisella tularemia* subsp. *novicida* (22.6% identity) could complement the intracellular survival defect of the C4-1 insertion mutant. C-terminal FLAG epitope-tagged versions of each allele were cloned and transformed into the C4-1 strain. Each complementing strain was used for infection of *A.c*. as in Figure 5D. Expression of the *L.longbeachae* allele completely restored intracellular survival of *L.p*. C4-1, whereas both the *Salmonella* and *Francisella* alleles failed to restore survival of the mutant (not shown).

**Figure 7:**
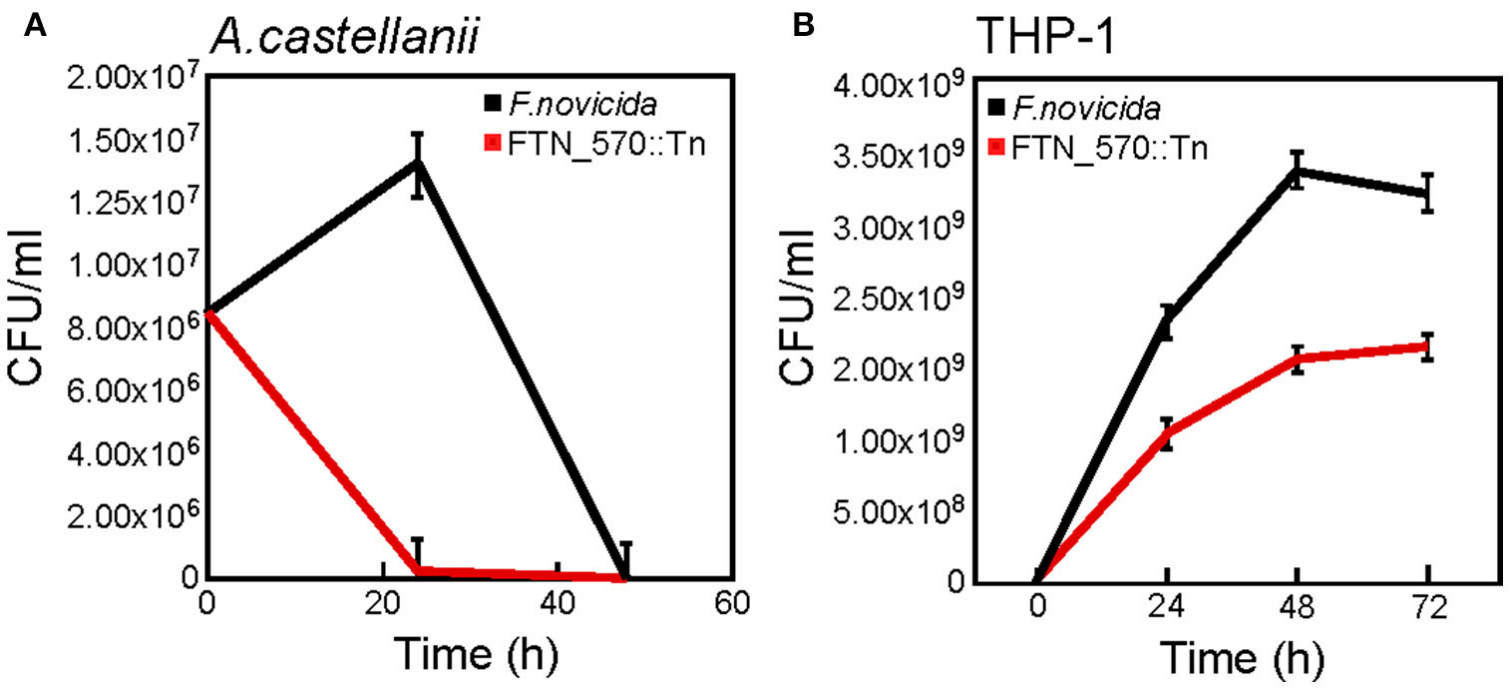
The *lpg0730* ortholog *FTN0570* from *Francisella tularensis* subsp. *novicida* is required for persistence/survival in *A.c*. and human THP-1 macrophages. **(A)** *F.novicida* WT (U112) or FTN0570 mutant (FTN0570::Tn) were used to infect *A.c.* cultures (MOI = 10), where host cells were lysed at indicated times and CFU/ml was determined by colony count after serial dilution and cultivation on supplemented TSA. **(B)** As in **(A)** except strains were used to infect differentiated THP-1 macrophages for indicated times prior to host cell lysis. Graphs indicate mean CFU/ml (three biological replicates) with standard deviations indicated.

Although the Lpg0730 (PerM) ortholog in *F.novicida* could not restore survival of *L.p*., we had access to a sequence-cataloged transposon library of *F.novicida* U112 in the laboratory (Provided by F. Heffron). The library contained an insertion mutant in the *perM* locus (FTN0570). We used this strain and WT *F.novicida* U112 for infections of either *A.c*. or THP-1 macrophages. We determined that WT *F.novicida* persists in *A.c*. for over 24 h, while the FTN0570 failed to colonize amoebae (Figure 7A). The FTN0570 insertion mutant was also significantly attenuated for pathogenesis of THP-1 when compared to WT *F.novicida* over a 72 h time-course of infection (Figure 7B). These observations highlight the conservation of Lpg0730-like ABC import complexes and their requirement for optimal host cell colonization.

## Discussion

Details regarding physiological requirements of *L.p*. during the intracellular life-cycle are only beginning to be elucidated. Further, identification and characterization of *L.p*. factors required for intracellular survival even in the presence of a functional Dot/Icm transporter are even more limited. When we designed this screen, we anticipated that an abundance of candidates would be identified from the Dot/Icm effector protein catalog (Zhu et al., 2011). Just as *dot/icm* is required for virulence, nearly all effectors described to date are dispensable. We hypothesized that the extremely large catalog of effectors in *L.p*. (~10% of the genome) was owed to particular sets being required for colonization of a particular host. The taxonomically diverse host-range of *L.p*. could highlight specificity of particular subsets of effectors for survival in a particular host cell. We chose *A. castellanii* as host for the initial phase of the screen as a natural environmental host. *A.c*. transitions from trophozoite to cyst and reverts based upon nutrient availability (Lloyd, 2014).

Owing to our hypothesis, we indeed found a degree of host-cell specificity associated with several candidates identified in the screen, however we were surprised that only one effector, SdhA had a transposon insertion in its ORF (Laguna et al., 2006; Creasey and Isberg, 2012). Intergenic regions surrounding Lem25 and RvfA were also interrupted, but multiple complementation attempts were unsuccessful (Huang et al., 2011). Both of these mutants were more attenuated for survival in *A.c*. than in any of the metazoan host cells examined, so should be explored with greater scrutiny in the future. We also identified 12 insertions in *dot/icm* encoding genes. While none of these mutants supported translocation of Cya-RalF in the adenylate-cyclase reporter assay (not shown), it was notable that eight of the insertions were found in the DotLMN type IV coupling protein sub-complex (Sutherland et al., 2012). This provides additional evidence that separates the initial stages of substrate recognition (DotLMN) from a superstructure that renders *L.p*. sensitive to NaCl via cytoplasm accessible conduit.

Amino acid availability to *L.p*. while in a phagosome was revealed as a key physiological constraint, similar to early studies using defined chemical media. *lpg2276* (Glu/Leu/Phe/Val dehydrogenase) was upregulated during intracellular replication in amoebae exclusively. Similarly *lpg1811* (Aspartokinase-diaminopimelate decarboxylase), important in lysine synthesis was highly upregulated in amoebae. The gene was determined dispensable in all metazoan cell types, both here and in a separate study where the *lpg1811* locus lost functionality over time when *L.p*. was continuously subjected to passage through a macrophage cell line (Ensminger et al., 2012). These results suggest that accessible lysine concentrations in the host must be distinct when comparing amoebae to macrophage. Other candidates identified in the screen can be directly correlated with particular environmental stressors encountered during colonization of protozoan cells. ProQm is a RNA-associated regulatory protein that governs activation of the ProP osmoprotectant transport system. Osmotic pressure changes are more likely to occur in the environmental host, especially in instances of desiccation upon encystment. HtpG (Hsp90) was found dispensable for survival in metazoan hosts, but was required for optimal infection of *A.c*., perhaps a result of elevated temperature used to accelerate *A.c*. infections.

The most striking result of the screen was the identification of three independent transposon insertions in the *lpg0730* locus. Each of the insertions rendered *L.p*. completely attenuated for survival in *A.c*. and THP-1 host cells. *lpg0730* and an additional candidate *lpg0122* were both annotated to encode ABC import complex proteins (Theodoulou and Kerr, 2015). We selected these *loci* for further characterization as the identified substrates of the transporter families (YdiK and NtrD/SsrB, respectively) are variable and often ill-defined experimentally. Both *lpg0730* and *lpg0122* were demonstrated to be required for survival even in the presence of a functional Dot/Icm transporter, highlighting the importance of acquisition of particular substrates during colonization of the host cell.

Additionally, both Lpg0730 and Lpg0122 were conserved in multiple human pathogenic species that are capable of colonizing protozoa. Here we demonstrated the PerM ortholog in *F. tularensis* subsp. *novicida* was required for persistence in *A.c*. and optimal pathogenesis in THP-1 macrophages. This result supports the notion that in order to colonize amoebae, these distinct import complexes must be functional, and their activities take precedence over the contribution of other virulence mechanisms. Identification of the imported substrates will be of critical importance in order to develop methods to prevent acquisition of the particular nutrients supplied by the amoebae host.

Because the transmission of *L.p*. to humans is confined to dispersal via water droplet (aerosols), outbreaks of legionellosis are normally confined to a single source that has been compromised with infectious bacteria (Burillo et al., 2017). The barricade for controlling disease spread therefore resides predominantly upstream of the pathogen-human interface. A variety of methods are used both commercially and municipally to eliminate water-borne pathogens, including elevating temperature of contained water, lowering pH, or flushing water lines with heavy metals (Zgonc and Baideme, 2015). These treatment methods are often effective for elimination of *L.p*. and other bacteria. Contrarily, these sterilization scenarios can be insufficient to eradicate natural host amoebae; many of which can differentiate to an environmentally resistant cyst-form as part of their life-cycle (Lloyd, 2014).

Planktonic *L.p*. can be found in multiple physiological states in freshwater. Viable bacteria, which can be free-living or associated with polymicrobial biofilms, may be detected via culture on artificial media. Indeed *L.p*. has been demonstrated to remain infectious to human macrophage cell lines at low levels even after incubation at low temperature (10–15°C) in freshwater medium (Fraquil) for 6 months (Mendis et al., 2015). A second form, viable but not culturable (VBNC) could represent an adaptation of the bacterium to the nutrient-depleted environment that is freshwater. The VBNC state appears to be induced upon introduction of environmental stressors, especially temperature above 70°C (Ducret et al., 2015). VBNC *L.p*. were shown to be incapable of colonizing human airway epithelia or macrophage cell lines, but could be resuscitated when introduced to *A. polyphaga*. After completion of their intracellular life-cycle in amoebae, these protozoan-primed VBNC were now infectious to alveolar epithelial and macrophage cell lines (Epalle et al., 2014).

These results, along with the aforementioned studies comparing the infectivity of *L.p*. cultivated in laboratory medium compared to protozoan-primed bacteria, suggest that the state of the bacterium encountered at the human interface is most likely the transmissive form generated during egress from protozoa, and not the form generated through *in vitro* culture in rich medium. We felt it imperative to more thoroughly investigate the *L.p*.-protozoan interface in order to identify genetic factors required for colonization and replication of *L.p*. Because replication of *L.p*. in freshwater is restricted to the confines of protozoa, these genetic targets could be exploited to block proliferation of *L.p*. and other pathogens in amoebae, and if used in concert with current sterilization practices, could significantly reduce exposure of protozoan-primed *L.p*., and other bacteria to humans.

## Materials and methods

### Bacterial strains, plasmids, media, and culture conditions

All work performed in this study was approved by the OHSU Institutional Biosafety Committee (protocol #08-53). Bacterial strains, plasmids, and primers used in this study are summarized in Table S2. *L.p*. strains were cultured in ACES buffered yeast extract (AYE) or on AYE agar plates (0.2% activated charcoal) (CYEA) at 37°C. Media was supplemented with the following antibiotics where appropriate: chloramphenicol (Cm); (6.25 μg/ml), kanamycin (Kan); (20 μg/ml), streptomycin (Str); (50 μg/ml). Sucrose (5% w/v) was included in CYEA for counter-selection of recombinant plasmid pSR47S. *Escherichia coli* strains were cultured at 37°C in Luria Bertani (LB) medium supplemented with Cm (25 μg/ml), Kan (50 μg/ml), Str (50 μg/ml) where appropriate. *F. novicida* strains were cultured in Tryptic Soy Broth (TSB) supplemented with 0.1% cysteine at 37°C.

### Cell culture

Axenic *A.c*. strain Neff (ATCC 30010) and *A. polyphaga* strain (Puschkarew) Page CCAP 1501/3b (ATCC 30872) cells were propagated in supplemented PYG medium (ATCC 712) at room temperature (RT), harvested and diluted into fresh medium twice weekly. *H. vermiformis* strain CDC-19 (ATCC 50237) was propagated in SCGYEM medium (ATCC 1021) at 30°C, harvested and diluted into fresh medium weekly. Murine macrophage J774.A1 (ATCC TIB-67), human monocyte THP-1 (ATCC TIB-202) and FcγRII transgenic—Chinese Hamster ovary (CHO) cells (C. Roy laboratory) were maintained at 37°C with 5% CO_2_. Undifferentiated THP-1 cells were cultured in suspension in RPMI medium supplemented with 10% fetal bovine serum (FBS). Forty-eight hours prior to infection, cells were differentiated in 24 or 96-well tissue culture plates, using phorbol 12-myristate 13-acetate (PMA). CHO FcγRII and J774.A1 were propagated in αMEM and DMEM respectively, supplemented with 10% FBS.

### *In vitro* growth studies

*L.p*. strains were inoculated in triplicate into 10 ml AYE or minimal media to achieve an initial OD_600 nm_ of 0.1 (Warren and Miller, 1979). Cultures were incubated on an orbital shaker (200 × rpm) at 37°C. One hundred microliters aliquots were collected from each culture at indicated times post-inoculation, suspended in 0.9 ml 1xPBS, and OD_600 nm_ were calculated via spectrophotometer.

### Transposon mutagenesis

*L.p*. JR32::*gfp* was generated via single-copy allelic integration of the *gfp*_*mut*3_ locus on to the chromosome immediately 3′ to the *wipA* (*lpg2718*) locus. A region encompassing sequential tandem *tac* and *icmR* promoters located 5′ to the *gfp*_*mut*3_ ORF was amplified from the plasmid pECR350 using primers 551/552. Flanking 1 kb fragments located 5′ and 3′ to the *wipA* stop codon were amplified using 549/550 and 553/554 respectively. The three PCR products were mixed (1:1:1) and used as template for short overlap extension (SOE) PCR using the 549/554 primer set. The resulting ~2.8 kb PCR product was ligated into the pSR47S vector linearized with *Bam*H1 using T4 DNA ligase (New England Biolabs). The resulting plasmid was designated pECL529. After sequence validation, pECL529 was transformed into *E. coli* DH5α λpir and introduced to JR32 via tri-parental mating scheme using *E. coli* DH5α, pRK600 as the helper strain. Transconjugants were generated through selection on CYEA Str, Kan. Colonies were isolated and plated on CYEA Str supplemented with 5% sucrose to force plasmid excision. Sucrose-resistant/kanamycin-sensitive isolates were examined using PCR to verify *gfp*_*mut*3_ insertion 3′ to the *wipA* locus. GFP positive isolates confirmed by fluorescence after growth in AYE broth supplemented with 1 mM IPTG. The resulting strain generated *JR32::gfp*_*mut*3_. The transposon-containing plasmid, pNH3503 was introduced into *JR32::gfp*_*mut*3_by electroporation. Twenty rounds of transformation and subsequent selection on CYEA Kan generated over 4,200 isolates which were randomly screened for the transposon insertion using the 573/574 primer set. Each individual insertion mutant was cultivated on CYEA in 96-well plates for 48 h at 37°C, re-suspended in stock solution (5% glycerol _W/V_, 2% peptone _W/V_), cataloged and stored at −80°C.

### Intracellular replication in protozoa and macrophages

Seventy-two hours quantitative intracellular growth assays were performed in a similar fashion to those previously described (Zamboni et al., 2003; Ninio et al., 2005). For the transposon mutagenesis screen, *A.c*. were suspended in supplemented ATCC 712 PYG media and cultured for 72 h, after which cells were harvested via centrifugation (400 × g) for 10 m. Media was aspirated and cells were suspended in infection media (3.4 mM Na citrate plus ATCC 712 salts) and seeded to tissue culture wells at 5 × 10^5^ (0.5 ml) or 1.25 × 10^5^ (0.125 ml) per well in 24 or 96-well format, respectively. Amoebae were allowed to adhere to plates overnight at RT. Host cells were infected with *L.p*. strains cultured to post-exponential phase in AYE broth (1 mM IPTG) at MOI = 10 (24-well) or ~10 (96-well). For the screening process, 96-well plates were floated on a 37°C water bath for 5 m and transferred to humidified incubator (37°C, 5%CO_2_) for 18 h. Intracellular growth was assessed qualitatively using light and fluorescence microscopy (AMG EVOS*fl*). J774.A1 were cultured to near-confluency, harvested by centrifugation, seeded (2.5 × 10^5^/well) in 24-well plates and incubated overnight at 37°C, 5%CO_2_. One hour prior to infection, cells were washed 3x with 1x PBS, 0.5 ml fresh DMEM (10% FBS) was added to each well, and plates were returned to incubator. THP-1 monocytes were cultured for 3–5 days in RPMI (10% FBS) prior to harvest by centrifugation. Cells were resuspended in RPMI (10% FBS, 100 ng/ml PMA), seeded (2.5 × 10^5^/well) in 24-well plates and incubated 48 h at 37°C, 5%CO_2_. One hour prior to infection, cells were washed 3x with 1x PBS, after which 0.5 ml RPMI (10% FBS) was added to each well. CHO FcγRII were cultivated in αMEM (10% FBS) and processed similar to J774.A1, with the exception being the inclusion of anti- heat-killed *L.p*. polyclonal antisera (Pacific Immunology) in the αMEM replacement media 1 h prior to infection. The *A.c*. and THP-1 infections with *F. novicida* were performed in a similar manner, using bacteria cultured to post-exponential phase in TSB 0.1% cysteine.

### DNA sequencing

Insertion mutants were sequenced either by PCR using purified genomic DNA as template for primer 780, or with “arbitrary” PCR modified from O'Toole and Kolter (1998). Briefly, the 1° PCR utilized purified gDNA as template for amplification with the 571/Arb1c primer set, followed by a 2° PCR using first PCR product as a template for amplification with the 780/781 primer set.

### Protein sequence alignment

*L.p*. reference amino acid sequences of polypeptides were derived from the Philadelphia 1 genome (NC_002942.5) National Center for Biotechnology Information (NCBI). Sequence alignments were performed using BLASTP with default parameters (NCBI/NLM). Phylogenetic analysis was performed by aligning *L.p*. proteins with individually selected bacterial species using BLASTP. Closest orthologous polypeptides from each species were aligned using Clustal Omega software (http://www.ebi.ac.uk/Tools/msa/clustalo/) European Molecular Biology Laboratory (EMBL).

### Cya translocation assay

Translocation assays were performed as described previously (Nagai et al., 2005; Ninio et al., 2005). Briefly, monolayers containing 1 × 10^5^ CHO FcγRII cells were infected with 3 × 10^6^ opsonized *L.p*. (MOI = 30) expressing Cya hybrid proteins. After 1 h incubation at 37°C, 5% CO_2_, monolayers were washed with 1x PBS and lysed. Total cAMP was extracted and quantified using cAMP Biotrak Enzymeimmumoassay System (GE Healthcare).

### Transcriptional analyses

To analyze transcription of *L.p*. genes during intracellular growth in *A. castellanii, A. polyphaga, H. vermiformis*, and J774.A1, cells were seeded in appropriate media under conditions as suited for each, and challenged with *L.p*. at an MOI = 200. The infection was synchronized by centrifuging the cells at 400 × g for 10 m and bacterial RNA was extracted immediately (t_0_) and 18 h (t_18_) post-infection. For extraction, infection buffer was removed and monolayers were disrupted with a sterile cell scraper. One milliliter of PBS was added to each well and vigorously mixed with pipette. Suspensions were transferred to a 14 ml conical tube and centrifuged for 10 m at 3,000 rpm. Supernatants were aspirated and pellets were re-suspended in 10 ml ice cold sterile H_2_O (protozoan) or 10 ml ice cold sterile H_2_O, 0.1% Triton X-100 (macrophage) and incubated on ice for 10 m. Lysates were centrifuged for 10 m at 4,000 × rpm. Supernatants were aspirated and pellets were resuspended in 1 ml guanidine thiocyanate buffer (GTC) (4 M guanidine thiocyanate, 0.5% Na N-lauryl sarcosine, 25 mM Na citrate, pH 7.0, 1 M β-mercaptoethanol) and transferred to a microcentrifuge tube. Suspensions were passed through a 21 gauge needle 3x and centrifuged 10 m at 15,000 × rpm. After aspiration, pellets were washed with 1xPBS, 0.1% Tween-20 and centrifuged an additional 10 m at 15,000 × rpm. After aspiration, pellets were re-suspended in 100 μl of 10 mg/ml lysozyme (Fisher Bioreagents) in 10 mM Tris, pH 8.0. Samples were incubated 15 m at RT. 750 μl of 65°C Trizol reagent (Invitrogen) was added to each sample, mixed and centrifuged for 10 m at 15,000 × rpm. The aqueous phase was transferred to a microcentrifuge tube and mixed with 500 μl of 100% ethanol. Samples were finally transferred to an RNeasy column and isolated according to manufacturer instructions, except that the in-column DNAse treatment was increased to 1 h (Qiagen). Purified RNA samples were analyzed with a Nano-drop *ND-1000* spectrophotometer and stored in aliquots at ^−^80°C.

Five Hundred nanograms of total bacterial RNA isolated from intracellular WT *L.p*. was subjected to cDNA synthesis using a Takara BluePrint RT reagent kit with random hexameric priming (Clontech) according to manufacturer instructions. After cDNA synthesis was performed in a thermocycler, samples were diluted 1:15 and stored on ice prior to qRT-PCR analysis. Primer sets were designed to target specific transcripts on the *L.p*. genome (Figure 4, Table S2). qRT-PCR samples were prepared using BioRad IQ SYBR Green Supermix. Reactions for each primer set probe were performed in triplicate in a Bio-Rad clear 96-well multiplate on a Bio-Rad Opticon thermocycler. Results were analyzed using Step One Software, v2.2. Relative expression, ΔΔCt analysis, was performed using *gyrB* as a reference transcript.

## Author contributions

Conceptualization, methodology, funding acquisition, and project administration (EC). Investigation, data curation, validation, and formal analysis (AL, SD, RJ, GR, and EC). Visualization and original draft preparation (AL, SD, and EC). Review and Editing (EC).

### Conflict of interest statement

The authors declare that the research was conducted in the absence of any commercial or financial relationships that could be construed as a potential conflict of interest.

## Supplementary material

Figure S1 Lpg0730 is conserved in multiple genera that colonize protozoa.**(A)** Clustal Omega (EMBL) phylogenetic cladogram depicting Lpg0730 (*L.p*.) and its relationship to homologous/orthologous polypeptide sequences found in the genera shown. **(B)** BLASTP amino acid sequence alignment (NCBI) of Lpg0730 (red) and homologous/orthologous polypeptide sequences found in the genera shown (multispecies). Nine representative transmembrane segments are shaded (gray). **(C)** BLASTP alignment of Lpg0730 and YdiK (*E. coli*). ^*^indicate conserved residues. Transmembrane segments are shaded (gray). **(D)** BLASTP alignment of Lpg0730 and PerM (FTN0570) from *F. tularensis subsp. novicida*. ^*^indicate conserved residues.

Figure S2 Lpg0122 is conserved in multiple genera that colonize protozoa.**(A)** Clustal Omega (EMBL) phylogenetic cladogram depicting Lpg0122 (*L.p*.) and its relationship to homologous/orthologous polypeptide sequences found in the genera shown. **(B)** BLASTP amino acid sequence alignment (NCBI) of Lpg0122 (blue) and homologous/orthologous polypeptide sequences found in the genera shown (multispecies). **(C)** BLASTP alignment of Lpg0730 and TauB (*E. coli*). ^*^indicate conserved residues.

Figure S3 Complementation of *lpg0730*::Tn with Lpg0730_*FLAG*_. Cultured *A.c*. were infected with indicated strain/plasmid combinations for 18 h. Representative live images were captured using light and fluorescence microscopy. Scale bar = 200 μm.
