# Supplementary material for: Identification of Conserved ABC Importers Necessary for Intracellular Survival of Legionella pneumophila in Multiple Hosts
Source: Front Cell Infect Microbiol. 2017 Nov 30;7:485. doi: 10.3389/fcimb.2017.00485 (PMC5714930; doi:10.3389/fcimb.2017.00485)
Supplement: Figure S1 — Lpg0730 is conserved in multiple genera that colonize protozoa.(A) Clustal Omega (EMBL) phylogenetic cladogram depicting Lpg0730 (L.p.) and its relationship to homologous/orthologous polypeptide sequences found in the genera shown. (B) BLASTP amino acid sequence alignment (NCBI) of Lpg0730 (red) and homologous/orthologous polypeptide sequences found in the genera shown (multispecies). Nine representative transmembrane segments are shaded (gray). (C) BLASTP alignment of Lpg0730 and YdiK (E. coli). *indicate conserved residues. Transmembrane segments are shaded (gray). (D) BLASTP alignment of Lpg0730 and PerM (FTN0570) from F. tularensis subsp. novicida. *indicate conserved residues. [file Image1.PDF]

# A

## Phylogenetic cladogram for Lpg0730

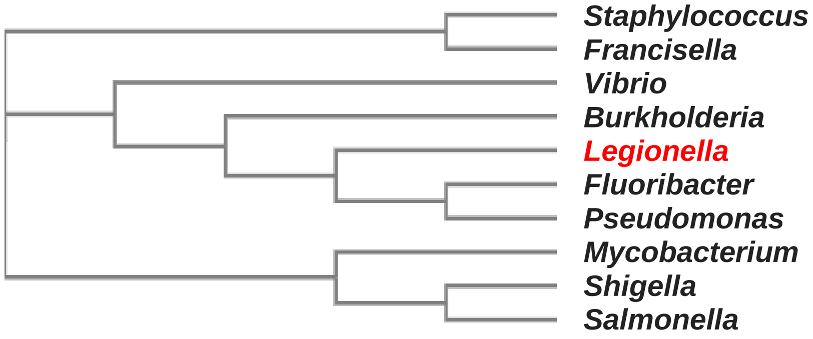


# B

## Multiple sequence alignment of *L.pneumophila* Lpg0730

```
Staphylococcus      ---MSSEENVKRQEKSKKKLNLNFPETRFMRFLGGKDLIFGLFMLILIGIVIFIFDQVSY
Francisella         ---------MT--------SYT-----------NIKK-------LSIIC---LVGALIGW
Vibrio              ---------MSEKIK----LAS-----------SHRV-------LVV--ALLASALACYW
Burkholderia        MGIPSRNENATDSAD----RTR-----------RFQR-------AASAGLYAVLVLIALY
Lpg0730             -------------------MNE-----------NHKE-------LISIGLTVSIVIFSLY
Fluoribacter        -------------------MNE-----------NHKE-------LISIGLTIGIVIISLF
Pseudomonas         ---------MAN---------------------NDR--------LLVQILLLALLGAALW
Mycobacterium       ---------MEKSQP----GYD-----------LPKL-------IFAVGAISILIVISFW
Shigella            ---------MVNVRQ----PRD-----------VAQI-------LLSVLFLAIMIVACLW
Salmonella          ---------MVNVRQ----PRD-----------IAQV-------LLSVLFLAIMIVACLW
                                                       :                       :

Staphylococcus      IFKPFIIVFNTIVAPIIVSLILYYLFNPIVNLM--ERYNISRLWG-VIILFLVIIGVISL
Francisella         VLYPFIYPILFA---GLLAIIL----APLQLYL--EQHIGRHKS--SFIIVIAILLCIFI
Vibrio              LVEPYINSIIMA---FIISLLM----FPIHDWF--EKKLPSHNNLAAFLSCVVLTVIIVI
Burkholderia        TARTFIPAVVWA---VVIAIAL----WPAFGWLERRPLFRKRHTLLAVLLTLAIGLLFVL
Lpg0730             IVHKFIPSMIWA---AIIVIAT----YPLYT--RWRKLFGNKHNTSAFLFTTLMGLLFLI
Fluoribacter        IIHRFIPSLIWA---SIIVIAT----YPLYE--RWRKFFGNKHNLSALLFTSLIGLLFIL
Pseudomonas         VMAPFISALLWG---AILAFAS----WPLMR--LLTRLLGGRETLAASILTTVWILVVAL
Mycobacterium       VLSPFLPGFVWA---GMIVIAT----WPVYT--AIRRRVRNSRWIAASVMVMLIILLFVF
Shigella            IVQPFILGFAWA---GTVVIAT----WPVLL--RLQKIMFGRRSLAVLVMTLLLVMVFII
Salmonella          IVQPFILGFAWA---GTIVIAT----WPVLL--KLQKILWGRRSLAVLVMTLLLVLLFVI
                        ::  .        : :       *                    :        . :

Staphylococcus      AINLLIPVISSQIKTFGTNFLHYITKVNQ--------FIDNITKYTVASNFYSQIQDYLN
Francisella         PLLVVISYVITEIISYLQHS-----ESLSQTFSQLSKSIANIPY--IGSTLQEHFDNLLN
Vibrio              PLLFIFGAIVQQGSKFSQNLYAWVTQGGIQALFNHPWVVKGLSL--ANHYL--PFE----
Burkholderia        PFGIVAGQTIDEA----HDMMRWFHDVLRTGIPM-PAFIEHLPT--GSEQAARWWHDNLA
Lpg0730             PLSWLIGILIKES----QLFINFLQHINKEGGAA-PEFFKNIPL--VGDDLIQYWDVNIG
Fluoribacter        PLSWLLGILIKEL----QIFINFLQSLNQQGGAA-PQFLKDFPF--IGNDLVTYWDNNIG
Pseudomonas         PLVWLGFNLADHV----RDATNFVRDVQLEGLPDAPEWVRGIPF--IGERLVNWWES-LD
Mycobacterium       PLALLINSIVENS----EPLMRWVKSPGQLTLPE-LYWLDSIPV--IGPKLFASWTTLVA
Shigella            PIALLVNSIVDGS----GPLIKAI-SSGDMTLPD-LAWLNTIPV--IGAKLYAGWHNLLD
Salmonella          PVALLVNSIVDGS----GPLIHAV-TGGDMALPD-LAWLNNIPL--VGAKLYAGWHSLLD
                     .  :                                 .  :

Staphylococcus      SLAKK----IPSMISDYFNGFGSK----VKNIAETVVNVGVFIVTTPFVLFFMLKDGHRF
Francisella         MVNQDKDI-IISNLGKILPT--------IRYIGFTSVSLVTDFLITLLLVYQFLVSSTSL
Vibrio              ---EISPQQIAQRIGQFATTFGSNLVSISAKILGDATSFVMHFFLMLFVPFFLLRDHDKI
Burkholderia        TPLESSPAV--KNLHS--APFVAM----TRHFGGRVVHGLVIFGFMLMTLFFIFQAGAKL
Lpg0730             KPGNIKEFL--SNLHVTLTPTSYY----IKQIGVNLAHRSFQVGFTLLTLFFFYRDGDKL
Fluoribacter        KPGMIKGFL--SNIHLSLAPTSYY----VKQVGSNLAHRSVQLGFTLLSLFFFYRDGDVL
Pseudomonas         QQGAALIASAKPYLGQVGNWFLAR----SAQIGSGV----LELTLSIVFVFFFYRDGPRL
Mycobacterium       SGGNAMMAKIQPYLGQGAAWFLTQ----LLSAGKFL----FHLAIMLLFSALLYLQGEEA
Shigella            MGGTAIMAKVRPYIGTTTTWFVGQ----AAHIGRFM----VHCALMLLFSALLYWRGEQV
Salmonella          MGGSAIMAKVRPYIGTTTTWFVGQ----AAHIGRFM----MHCALMLLFSALLYWRGEQV
                                 :                                 .    :
```

```
Staphylococcus      KDFSTKIVPPKFRKDYHDLLDKMSVQVGSYIQGQIIVSLCIGVLLFIGYSIIGLDYGLIL
Francisella         EKFLKKIVLKDF-HDSDSFISAAIATTRRVSLAIFLTAMLVGTIMTITFSMVGIPSPILF
Vibrio              ISAIRHILPLS-RSQEDRLLSEIENVSKSAVMGSFLTAIAQGVAGGIGMWLAGFPGLFWG
Burkholderia        GRQMLAGSSRAFGADGAALLQRMADAVRSTVVGLVVVGLGEGALLGIAYAVTGVPHATLL
Lpg0730             LLQIQHIGEYCLGDRWFRYSDRLPSALRGTVNGTIVVGIGVGILMGVCYALVGFPAPTLT
Fluoribacter        LSQIYQVGEYCLGKRWFRYADRLPRALRATVNGTIVVGLGVGILMGICYGLVGFPAPTLV
Pseudomonas         AAFVQRLLQRLVGERAEYYIDLVAGTVQRVVNGVIGTAAAQGLLALIGFLIAGVPGAIVL
Mycobacterium       MRGFRHFALRLAGMRGDAAVLLAAMSVRAVALGVVVTALTQAIIGGLGLGLAGVPFAAIL
Shigella            AQGIRHFATRLAGVRGDAAVLLAAQAIRAVALGVVVTALVQAVLGGIGLAVSGVPYATLL
Salmonella          AMGIRHFACRLAAKRGDAAVLLAAQAIRAVALGVVVTALVQAVLGGVGLAISGVPYATLL
                                                    . . ..   .    :   : *.

Staphylococcus      ACIAAVTSVVPYIGPTIAISPAIIIALITSPIMLLKLIVVWTA-VQFIEGHFISPNIMGK
Francisella         GFIAAIASMVPFMVGIIYILIGASVFVIYGAT-KAIIILIIGFSLNIFTDNIMQPKIINK
Vibrio              TMMGFASFIPVVGTALIWIPATLYLFLTGDTT-WAIFLAIWSVAVVGSIDNLLRPFLMQG
Burkholderia        GMLTAVAAMLPFCAPIVFLGSALWLLAQGSTV-AAVGVAIFGLVVVFVAEHFVRPVLIGG
Lpg0730             GFITALAAMIPFVVPVVFIIVALILLSVGSLI-GGIIVLVWGTLVMFVADHFVKPVLIGG
Fluoribacter        GFITALAAMIPFVVPIVFITVAIILFSFGSMI-GAIVVLVWGTLVMFVADHFVKPALIGG
Pseudomonas         GLVTFMLSLIPMGPPLAWIPATGWLVWKGEYG-MAVFLGIWGTFIISGVDNVLKPYLISR
Mycobacterium       TVLIFICCVAQLGPLLIMIPSIIYLFWTGDTT-WAVVMIVWAG-IVATMDGVLRPYLIKM
Shigella            TVLMILSCLVQLGPLPVLIPAIIWLYWTGDTT-WGTVLLVWSG-VVGTLDNVIRPMLIRM
Salmonella          TVVMILSCLVQLGPLPVLIPAIIWLYWTGDTT-WGTVLLVWSA-VVGTLDNVIRPVLIRM
                      :     :         :     :            : :    :      .: * ::

Staphylococcus      TLQIHPLTIIFILLSAGNLLGVVGVILGIPAYAILKVLVSHLYFLYKRRYNKYYGDDAGE
Francisella         QVKLSFVASLIGIMGGIHAFGFIGIFLGPVIFNVAFVGIEKLM-----NNQEY-------
Vibrio              SAGMNTLMIFFSLLGGIQLFGLIGLIYGPLIFAITMVLFNIYE-----EEFRSFLDQQDR
Burkholderia        SSRLPFLLVLFGILGGAETFGLIGLFIGPALMTILVVLWTDWV-----RA----------
Lpg0730             AIQLPFLAVLFGILGGVETLGLLGLFLGPMVMVLFVTLWQEPQ-----GHKKSSG-----
Fluoribacter        AIELPFLAVLFGILGGVETLGLLGLFVGPLVMVLFMTLWQEPQ-----ILVAAKKHPAC-
Pseudomonas         GGNLPLVIVLLGVFGGLIAFGFIGLFIGPTLLAVGYSLLLDWS-----RHSGQVQR----
Mycobacterium       GADLPMVLILTGVIGGMLSLGMIGLFIGPVVLAVSYTLLKAWM-----NDVPKPSDDLTE
Shigella            GADLPLILILSGVIGGLIAFGMIGLFIGPVLLAVSWRLFATWV-----EEVPPPTDQPEE
Salmonella          GADLPLLLILSGVIGGLIAFGMIGLFIGPVLLAVTWRLFSAWV-----HEVPAPTNEPEE
                       :  :  :  ::..   :*.:*:: *     :

Staphylococcus      YEFKNDEQEYIRD--
Francisella         ---------------
Vibrio              S--------------
Burkholderia        ---------------
Lpg0730             ---------------
Fluoribacter        ---------------
Pseudomonas         ---------------
Mycobacterium       VRAYMEEQLSEKE--
Shigella            ILEELGEIEKPNK--
Salmonella          ILEELDEIEEANKQS
```

# C

## Sequence alignment of Lpg0730 (*L.pneumophila*) and YdiK (*E.coli*)

```
Lpg0730    ------MNENHKELISIGLTVSIVIFSLYIVHKFIPSMIWAAIIVIATYPLYTRWRKLFG
   YdiK    MVNVRQPRDVAQILLSVLFLAIMIVACLWIVQPFILGFAWAGTVVIATWPVLLRLQKIMF
                  .:  : *:*: :  . ::: .*:**: ** .: **. :****:*:  * :*::

Lpg0730    NKHNTSAFLFTTLMGLLFLIPLSWLIGILIKESQLFINFLQHINKEGGAAPEFFKNIPLV
   YdiK    GRRSLAVLVMTLLLVMVFIIPIALLVNSIVDGSGPLIKAISSGD-MTLPDLAWLNTIPVI
            ::. :.:::* *: ::*:**:: *:  ::. *  :*: :.  :         :::.**::

Lpg0730    GDDLIQYWDVNIGKPGNIKEFLSNLHVT-LTPTSYYIKQIGVNLAHRSFQVGFTLLTLFF
   YdiK    GAKLYAGWHNLLDMGGTAIM--AKVRPYIGTTTTWFVGQA-AHIGRFMVHCALMLLFSAL
           * .*   *.  :   *.     ::::    * *:::: *  ..:.:  .: .: **   :

Lpg0730    FYRDGDKLLLQIQHIGEYCLGDRWFRYSDRLPSALRGTVNGTIVVGIGVGILMGVCYALV
   YdiK    LYWRGEQVAQGIRHFATRLAGVRGDAAVLLAAQAIRAVALGVVVTALVQAVLGGIGLAVS
           :*  *:::   *:*:.    * *          .*:*... *.:*..:  .:* *:  *:

Lpg0730    GFPAPTLTGFITALAAMIPFVVPVVFIIVALILLSVGSLIGGIIVLVWGTLVMFVADHFV
   YdiK    GVPYATLLTVLMILSCLVQLGPLPVLIPAIIWLYWTGDTTWGTVLLVWSGVVG-TLDNVI
           *.*  **  .:  *:.:: :    *:* . : *  .*.   * ::***. :*  . *..:

Lpg0730    KPVLIGGAIQLPFLAVLFGILGGVETLGLLGLFLGPMVMVLFVTL---WQEPQGHKKSS-
   YdiK    RPMLIRMGADLPLILILSGVIGGLIAFGMIGLFIGPVLLAVSWRLFAAWVEEVPPPTDQP
           :*:**  . :**:: :* *::**: ::*::***:**:::.:   *   * *     ...

Lpg0730    -------G-------
   YdiK    EEILEELGEIEKPNK
                  *
```

# D

## Sequence alignment of Lpg0730 (*L.pneumophila*) and PerM (*F.novicida*)

```
Lpg0730    ------------MNENHKELISIGLTVSIVIFSLYIVHKFIPSMIWAAIIV-IATYPLYT
PerM       MILKTIKDWYQNRYQNNEPIVFVGLMLF-----FYLVLTFLGDYIAPILAALVIAYLLDT
                          :*:: :: :** :      :*:* .*: . *   : . : :* * *

Lpg0730    RWRKLFGNKHNTSAFLFTTLMGLLFLIPLSWL----IGILIKESQLFINFLQHINKEGG-
PerM       -LVNILQKFTKLKRIVLVYIVYILFLIALLSLIFVLLPIIINQLIDFVKQASHILSSLKT
             ::: :  : . :::. :: :**** *  *     : *:*::   *::  .** ..

Lpg0730    AAPEFFKNIP-LVGDD---LIQYWDVNIGK---PGNIKEFLSNLHVTLTPTSYYIKQIGV
PerM       SLEELSIKYPTILTEDRINSIVSWFDSIDWKKISSNVGSFILQNTATTLPVLFS------
           :  *:  : * :: :*     *  *  .*.     .*: .*: :  .*  *. :

Lpg0730    NLAHRSFQVGFTLLTLFFFYRDGDKLLLQIQHIGEYCLGDRWFRYS---DRLPSALRGTV
PerM       ---V-LIYLFLVPLMVFYFLKDKEKMINWFKSFLPEENGALYYVWNDLKPKLADYVRGKA
                : : :. * :*:* :* :*:: :: :      *  :: :.    :*  . :**..

Lpg0730    NGTIVVGIGVGILMGVCYALVGFPAPTLTGFITALAAMIPFVVPVVFIIVALILLSVGSL
PerM       IELIIVST--LTYIGFAYFNLNYA--VLLAVGVGLSVIIPYVGMVIITIP---VIMVGIL
             *:*.      :*..*  :.:   .* .. ..*:.:**:*  *:: *    :: ** *

Lpg0730    IGGIIVLVWGTLVM-----FV----ADHFVKPVLIGGAIQLPFLAVLFGILGGVETLGLL
PerM       QFGL----NGTFVSMLIVFFVIQALDGNLLVPLLFSEVLDMHPVGVVSAILIFGGIWGLW
            *:     **:*       **     .::: *:*:. .::: :.*: .**       **

Lpg0730    GLFLGPMVMVLFVT---LWQEP-QGHKKSSG----
PerM       GIFFAIPLGLLFISGVNMFRNHLNGKKTQADINLC
           *:*:.  : :**::   ::::  :*:*..:.
```
