# Supplementary material for: Identification of Conserved ABC Importers Necessary for Intracellular Survival of Legionella pneumophila in Multiple Hosts
Source: Front Cell Infect Microbiol. 2017 Nov 30;7:485. doi: 10.3389/fcimb.2017.00485 (PMC5714930; doi:10.3389/fcimb.2017.00485)
Supplement: Figure S2 — Lpg0122 is conserved in multiple genera that colonize protozoa.(A) Clustal Omega (EMBL) phylogenetic cladogram depicting Lpg0122 (L.p.) and its relationship to homologous/orthologous polypeptide sequences found in the genera shown. (B) BLASTP amino acid sequence alignment (NCBI) of Lpg0122 (blue) and homologous/orthologous polypeptide sequences found in the genera shown (multispecies). (C) BLASTP alignment of Lpg0730 and TauB (E. coli). *indicate conserved residues. [file Image2.PDF]

A

Phylogenetic cladogram for Lpg0122

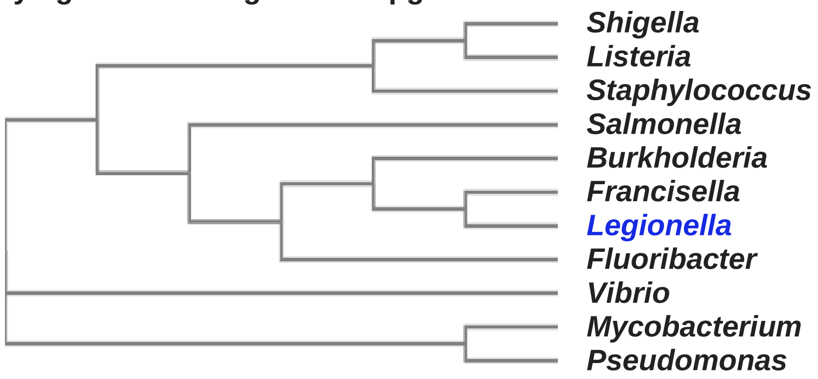

B

Multiple sequence alignment of *L.pneumophila* Lpg0122

```
Shigella          ----------------------MAIKLEIKNLYKIFGEHPQRAFKYIEQGLSKEQILEKT
Listeria          -----------------------MSKIKVEELTKIFGKKASKASSLLSQGKSKTDILKET
Staphylococcus    -------------------------MIKIQQLQHHFG-----------------------
Salmonella        -------------------MKSSQLVLEVKNVSKFFQKDRKQ------------------
Burkholderia      MEN---IIQTIPADTLQSSPRTGTQVLNIEHVSRGFDKNQ-S------------------
Francisella       ---------------------MTKKIFTVEKVNKEFNIKGGH------------------
Legionella        ---------------------MPETIINIENLSKSFKKAPSQ------------------
Fluoribacter      ---------------------MSETIIAIEGLRKSFKKAAEQ------------------
Vibrio            ----------------------MQPFVSFKNIGHTYQSDKQ-------------------
Mycobacterium     ----------------MGKSEPNKAHVLIEGVSKTFELRGGG------------------
Pseudomonas       MRATSLSILTSEHSLPVAAPADSRLEIEFRGVAKHFPGRGKSA-----------------
                                            . .. : : :

Shigella          GLSLGVKDASLAIEEGEIFVIMGLSGSGKSTMVRLLNRLIEPTRGQVLIDGVDIAKISDA
Listeria          GATIGVNKASFSVEEGEIFVIMGLSGSGKSTLVRLLNRLIEPTSGKIWLDGKELSSLNKK
Staphylococcus    -SHKVIHNFNLDISKGEIVTFIGKSGCGKSTLLNIIGGFIHPSSGRVIIDNEIKQQ----
Salmonella        -KVTVLEHINIEVTEGEIVALLGRSGSGKSTLLRMVAGLVEPDLGSIVCCDLPVNG----
Burkholderia      -ELLVLDDVNLTLHEGEIVGLLGRSGSGKSTLLRIISGLIRPTSGNVTYQGKPLDG----
Francisella       -SLKVLDNINFTLYEGEIVALLGKSGSGKSTLLRIIAGLLSPTSGDVLYRGKKVSA----
Legionella        -HLLVLEDVNFKLQEGEIVALLGKSGSGKSTLLRIIAGLIAPSSGTVTYRGKPVTR----
Fluoribacter      -NLLVLEDVNFKLQEGEIVALLGKSGSGKSTLLRIIAGLIAPTSGTVTYRGKPVTR----
Vibrio            -SVTVLNDVNFNVNKHEFVAIVGPSGCGKSTLLRLLSGLISPTEGEISVFGQPVTE----
Mycobacterium     -TTNALSPIDATVGQGRFISIVGPSGCGKSTLLNIVAGLVKPTTGAVSVDGEPVRG----
Pseudomonas       -ATLAVQGLDLAIRRGEVVSIIGPSGCGKSTLLNMGAGLYAPSEGEVRVGGERVSG----
                       :   .  : . ... ::* **.****::.:   :  *  * :   .

Shigella          ELREVRRKKIAMVFQSFALMPHMTVLDNTAFGMELAGI-NAEERREKALDALRQVGLENY
Listeria          ELLEVRRKSMSMVFQNFGLFPNRTINRNVEYGLEIQGM-DKEEREKNAAESLALVGLAGY
Staphylococcus    -----PSPDCLMLFQHHNLLPWKTINDNIRIGFQQKIS---DE---EINAQLKLVDLEGR
Salmonella        -----PGKNTSMVFQSFALFPWLNVFDSVAFGLQAQGI-SSDEVGKRTMDMLDLIGLSGY
Burkholderia      -----PAEGVAMVFQTFALFPWLTVLQNVEAGLEALGV-SVDERRTRALAAIDLIGLDGF
Francisella       -----PVPDISMVFQSFALMPWLTVLQNVELGLEARKI-SLEERRQRALKAIDMVGLDGF
Legionella        -----PVEGIAMVFQSFALMPWLTVLENVELGLEAQGI-SREERRHRAIEAIDIIGLDGF
Fluoribacter      -----PVDGIAMVFQSFALMPWLTVLENVELGLEAQGV-GREERRHRAIEAIDIIGLDGF
Vibrio            -----PREDIGIVFQKPTLLPWKNILDNVLFPLQHKFNYVSQKEKKHAEELLNTVGLSGF
Mycobacterium     -----PRRSIGVVFQEDSTLPWRTVMDNVRLGLEVEGM-RKAAQISRAKEMIDLVGLTGF
Pseudomonas       -----PVRKVSFMLQKDLLMPWRSIRRNIELGLEIDGR-AAGERRAIAEEMLEKCHLAGF
                             .::*    :*  .:  .    ::                 :    * .

Shigella          AHSYPDELSGGMRQRVGLARALAINPDILLMDEAFSALDPLIRTEMQDELVKLQAKHQ--
Listeria          GDQYPSQLSGGMQQRVGLARALANNPDILLMDEAFSALDPLNRKDMQDQLLDLQDKMK--
Staphylococcus    GKHFPEQLSGGMKQRVALCRAHVHKPNVILMDEPLGALDAFTRYKLQDQLVQLKHKTQ--
Salmonella        EKAYPRELSGGMRQRVGFARALAVEPDLLLMDEPFSALDIFTGNKLRQDLIELWENRVIK
Burkholderia      ENAYPRELSGGMRQRVGFARALVVDPTLLLMDEPFSALDVLTAETLRTDLLDLWTQGRMP
Francisella       ENAYPKELSGGMKQRVGFARALVLEPDVLLMDEPFSALDILTAENLREDLLDLWENND-A
Legionella        ESAFPKELSGGMRQRVGFARALVINPDVLLMDEPFSALDVLTAENLKSDLLELWKEKKTN
Fluoribacter      ESAFPKELSGGMRQRVGFARALVINPDVLLMDEPFSALDVLTAENLKSDLLELWKEKKTN
Vibrio            ETSMPNQLSGGMQQRVGIARALLLNPDILIMDEPFSALDALTREEMGFELQKIWMEKP--
Mycobacterium     EHAYPRELSGGMRQRVAIARTLALDPSVLLMDEPFGALDPQTRLMIGVELLRMWQSTS--
Pseudomonas       AEHYPFQLSGGMRQRAALARTLATDPQVLFLDEPFSALDAQTKMILQQDLARMLCEQR--
                      * :*****:**..:.*:   .* ::::** :.***      :  :*  :  .
```

```
Shigella            -RTIVFISHDLDEAMSIGDRIAIMQNGEVVQVGTPDEILNNPANDYVRTFFRGVDISQVF
Listeria            -KTIIFITHDLDEALRIGDHIMIMRDGSVVQTGSPEEILAHPANEYVEKFIEDVDRSKVY
Staphylococcus      -STIILVTHDIDEAIYLSDRIVLLGEG-GNIISQYEITASHPRSRNDS----------HL
Salmonella          TRSMLLVTHSVEEAVMLSDRVCLLGGNPSTITDIFDINIDRSQRIRENT-------SPVV
Burkholderia        IKSVLIVTHNIEEAVFMCNRILVLSSNPGRVVAETVVPFAHPR---NRL-------DPAF
Francisella         MKGILYVTHSIEEAVLTADRIIIFGSNPGFIRGELKINIPHPR---SSQ-------DPVV
Legionella          TNGILLVTHNIEEAATLADRIVIFGNDPGYIRAELPVTLPQPR---DPE-------SPEY
Fluoribacter        TNGILLVTHNIEEAATLADRIVIFGNDPGYIRAELPVTLPQPR---DPE-------SPEF
Vibrio              -KTVLFITHSISEAVLLADKVLVMGPRPSTVLEEIHIDLPRPRDIHTLQD------L-TF
Mycobacterium       -KTVLFVTHDIQEALLLSQEVWVMSYRPATISERLPVPFEYPRGPELLS-------TDDY
Pseudomonas         -KTALFITHDLVEAIAMSDRILVMSARPGTIVEEIEVGLPLRDNPLERRK------LPEI
                        : ::*.: **    :.: ::

Shigella            SAKDIARRTPNGLIRKTPGFGPRSALKLLQDEDR--EYGYVIERGNKFVGAVSIDSLKTA
Listeria            TASNVMIRPE---IVNFEKDGPRVALKRMREAGT--SSVFVVKRNRELVGIVHAAEVSKL
Staphylococcus      L-------KIRNEIMETFALN-----------HHQVEPEY-------YL-----------
Salmonella          D-------KISDTLNDRIASCA--------------------------------------
Burkholderia        R-------ALVDEIYAKMTARPTGAAA-----KRELEPGS-------WLPNVSTNLMAGL
Francisella         A-------DLVDQVYRMMTTAQTKELTERMNKKTAMTIGY-------RLPDVDISEMNGL
Legionella          L-------ALVDKIYTLMTTGPK-EKAKRAQRERQIGLGY-------RLPDVEPSELSGL
Fluoribacter        R-------ALVDKIYTLMTTGPK-EKAKRAQRERQIGLGY-------RLPDVEPSELTGL
Vibrio              G-------SYTNKIREYFYRSERQNCAESQDEGNVEPIRR-------TVA----------
Mycobacterium       Q-------HLNARIWSVLQKESQRAFDE---REHLLSSR---------------------
Pseudomonas         G-------PLVGRLMTLLKVGESAELH---------------------------------
                                 :

Shigella            LTQ------QQGLD--AALIDAPLAVDAQTPLSELLSHVGQAPCAVPVV----------D
Listeria            VKEN-----ITSLE--TALHRDVPTTGLDTPLAEIMDTISTTTIPI-AV----------T
Staphylococcus      ------------------------------------------------------------
Salmonella          ------------------------------------------------------------
Burkholderia        IETLAAAPYNGRADMPEIARTLQLEVDDLFPIAEMLQYLGFAEISEGDVFLTPPAQRFAE
Francisella         LDEMAEIKNVEAVDLPQLADDLHLDINDLFPIIEILSILRFAEVSDGDIKMTAMGRKFID
Legionella          IETMKSFE--ERIDLPELADELMMNIDDLFPILETLEILGFAKVSAGDIQLSELGKQFSE
Fluoribacter        IETMTSFE--ERIDLPELADELMMNIDDLFPILETLEILGFAKVSAGDIQLSDLGKQFAE
Vibrio              ------------------------------------------------------------
Mycobacterium       ------------------------------------------------------------
Pseudomonas         ------------------------------------------------------------

Shigella            ------------------------------------------------------------
Listeria            ------------------------------------------------------------
Staphylococcus      ------------------------------------------------------------
Salmonella          ------------------------------------------------------------
Burkholderia        GSTQERKIMFADHLLQSVPLAARIRKVLNERPGHRAPRVRFEQELEDMLSDSAAQETLDA
Francisella         SNIDERKFIFGRLFLKYIPLARHVVKVLSERESQSAPRSRFLAELDDYYPMDVAERVFDT
Legionella          ADLQERKQLFAQRLLEKVPLARYIRRVLDEKAGHRVSEERFLSKLEDYLSEKEADRVLKT
Fluoribacter        ADLQERKKLFAQRLLEKVPLARHIRRILDEKVGHRVSEERFLSKLEDYLSEKEADRVLRT
Vibrio              ------------------------------------------------------------
Mycobacterium       ------------------------------------------------------------
Pseudomonas         ------------------------------------------------------------

Shigella            --EDQQYVGIISKGMLLRALDREGVNNG-----
Listeria            --EDGKLKGIIIRGSVLAALSGNEVNVNA----
Staphylococcus      ---------------------------------
Salmonella          ---------------------------------
Burkholderia        VIDWGRYAEIFSYNDKTETFSLADVET------
Francisella         FIDWARYAELIYYDANTGVISLDENAAEYIKKM
Legionella          MIDWGRYAEIFAYDFNTGILSLENPGKGA----
Fluoribacter        MIDWGRYAEIFAYDFNTGILSLENPGKGL----
Vibrio              ---------------------------------
Mycobacterium       ---------------------------------
Pseudomonas         ---------------------------------
```

C

Sequence alignment of Lpg0122 (*L.pneumophila*) and TauB (*E.coli*)

```
Lpg0122      MPETIINIENLSKSFKKAPSQHLLVLEDVNFKLQEGEIVALLGKSGSGKSTLLRIIAGLI
TauB         ----MLQISHLYADYGG-----KPALEDINLTLESGELLVVLGPSGCGKTTLLNLIAGFV
                 :::*.:*  .:         .***:*:.*:.**::.:** **.**:***.:***::

Lpg0122      APSSGTVTYRGKPVTRPVEGIAMVFQSFALMPWLTVLENVELGLEAQGISREERRHRAIE
TauB         PYQHGSILLAGKRIEGPGAERGVVFQNEGLLPWRNVQDNVAFGLQLAGIEKMQRLEIAHQ
               . *::   ** :  *    .:***. .*:** .* :** :**:  **.: :* . * :

Lpg0122      AIDIIGLDGFESAFPKELSGGMRQRVGFARALVINPDVLLMDEPFSALDVLTAENLKSDL
TauB         MLKKVGLEGAEKRYIWQLSGGQRQRVGIARALAANPQLLLLDEPFGALDAFTRDQMQTLL
              :. :**:* *. :  :**** *****:****. **::**:****.***.:* ::::: *

Lpg0122      LELWKEKKTNTNGILLVTHNIEEAATLADRIVIFGNDPGYIRAELPVTLPQPRD------
TauB         LKLWQETG---KQVLLITHDIEEAVFMATELVLLSSGPGRVLERLSLNFARRFVAGESSR
             *:**:*.    : :**:**:****. :* .:*::...** :  .* :.: :

Lpg0122      --PESPEYLALVDKIYTLMTTGPKEKAKRAQRERQIGLGYRLPDVEPSELSGLIETMKSF
TauB         SIKSDPQFIAMREYVLSRV----------------------------------FEQREAF
                ..*:::*: : : : :                                  :*  ::*

Lpg0122      EERIDLPELADELMMNIDDLFPILETLEILGFAKVSAGDIQLSELGKQFSEADLQERKQL
TauB         S-----------------------------------------------------------
             .

Lpg0122      FAQRLLEKVPLARYIRRVLDEKAGHRVSEERFLSKLEDYLSEKEADRVLKTMIDWGRYAE
TauB         ------------------------------------------------------------


Lpg0122      IFAYDFNTGILSLENPGKGA
TauB         --------------------
```
