# Supplementary figures and images for: Identification of Conserved ABC Importers Necessary for Intracellular Survival of Legionella pneumophila in Multiple Hosts
Source: Front Cell Infect Microbiol. 2017 Nov 30;7:485. doi: 10.3389/fcimb.2017.00485 (PMC5714930; doi:10.3389/fcimb.2017.00485)

*A. castellanii*  
MOI=10, 18h post-infection

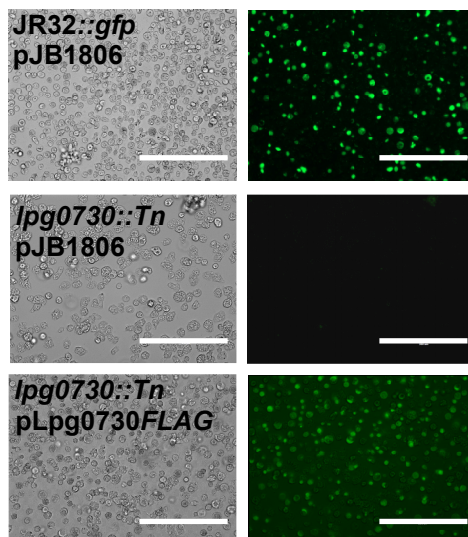

Supplement: Figure S3 — Complementation of lpg0730::Tn with Lpg0730FLAG. Cultured A.c. were infected with indicated strain/plasmid combinations for 18 h. Representative live images were captured using light and fluorescence microscopy. Scale bar = 200 μm. [file Image3.PDF]
