# Supplementary material for: Identification of Conserved ABC Importers Necessary for Intracellular Survival of Legionella pneumophila in Multiple Hosts
Source: Front Cell Infect Microbiol. 2017 Nov 30;7:485. doi: 10.3389/fcimb.2017.00485 (PMC5714930; doi:10.3389/fcimb.2017.00485)
Supplement: Supplementary file 4 [file Table1.PDF]

Supplemental Table 1.

Candidates identified defective for survival in *A.castellanii*

Isolate	Intracellular survival (approx. % of WT)
A10-20	20
B9-21	20
B10-21	20
C8-34	20
D1-1	50
F6-18	50
F8-16	50
F12-18	50
H1-15	20
H3-29	20
H9-15	50
H10-15	50
H11-21	20
H12-15	20
