# Supplementary material for: Identification of Conserved ABC Importers Necessary for Intracellular Survival of Legionella pneumophila in Multiple Hosts
Source: Front Cell Infect Microbiol. 2017 Nov 30;7:485. doi: 10.3389/fcimb.2017.00485 (PMC5714930; doi:10.3389/fcimb.2017.00485)
Supplement: Supplementary file 5 [file Table2.PDF]

Table S2. Bacterial strains, plasmids, and primers employed in this study

Bacterial Strains	Genotype	Reference
L.pneumophila		
JR32	Philadelphia 1, serogroup 1 *rpsL*	[1]
JR32::*gfp*	JR32::P*tac*, P*icmR*, gfp_{mut3}	This study
JR32Δ*dotA*::*gfp*	JR32::*gfp* Δ*dotA*	This study
C4-1	JR32::*gfp lpg0730*::TnNH3503	This study
D1-37	JR32::*gfp lpg0122*::TnNH3503	This study
Δ*lpg0122*	JR32::*gfp* Δ*lpg0122*	This study
CR39	Philadelphia 1, serogroup 1, strain LP01 *rpsL*	[2]
CR58	LP01 Δ*dotA*	[2]
CR157	LP01 Δ*icmW*	[3]
CR393	LP01 Δ*icmS*	[4]
CR503	LP01 Δ*icmS*,Δ*icmW*	[4]
F.tularensis subsp. *novicida*		
U112		[5]
FTN0570	U112, FTN0570 (*perM*)::Tn	This study
E.coli		
DH5α	*supE44* Δ*lacU169 (*Φ80*lacZ*Δ*M15) hsdR17 recA1 endA1 gyrA96 thi-1 relA1*	[6]
DH5α λpir	*supE44* Δ*lacU169 (*Φ80*lacZ*Δ*M15) hsdR17 recA1 endA1 gyrA96 thi-1 relA1* λ(*pir*)	[7]
MC1061	K-12 F^- λ^- Δ*(ara-leu)7697* [*araD139*]B/r Δ*(codB-lacI)3 galK16 galE15* $e14^-$ *mcrA0 relA1 rpsL150*(Str^R) *spoT1 mcrB1 hsdR2*(r^-m^+)	[8]

Plasmids	Properties	Primers	Reference
pAM239	pMMB207.gfp_{mut3}		[9]
pCya	pMMB207NT.*cya*		[10]
pCya-RalF	pMMB207NT.*cya.ralF*		[10]
pEC35	pMMB207NT.*cya.sidG*		[7]
pEC350	pJB1806.gfp_{mut3}	220,301	This study
pECG622	pJB1806.*lpg0122*	1048,1049	This study
pECL509	pGEMT.1000bp 5'($wipA_{TGA}$).P*tac*.P*icmR*.gfp_{mut3}.1000bp 3' ($wipA_{TGA}$)	See Methods	This study
pECL529	pSR47S.1000bp 5'($wipA_{TGA}$).P*tac*.P*icmR*.gfp_{mut3}.1000bp 3' ($wipA_{TGA}$)	See Methods	This study
pECL530	pJB1806.*lpg0730*	1054.1055	This study
pECL601	pJB1806.*perM* (*S.enterica*)*FLAG*	1072,1073	This study
pECL602	pJB1806.FTN0570 (*F.novicida*)*FLAG*	1058,1069	This study
pECL603	pJB1806.*lpg0730FLAG*	1054,1068	This study
pECL623	pJB1806.*llo0829* (*L.longbeachae*)*FLAG*	689,690	This study
pJB1806	RSF1010-derived Δ*oriT* (*cat*) P*tac*		*J.P.Vogel*
pNH3503	ColE1*ori* P*lac.tpase bla rpsL mini-minimariner* (*npt2*)		[8]
pSR47S	R6K*ori npt2 sacB*		[11]

Primer	Sequence	Site
Arb-1c	GGCCAGCGAGCTAACGAGACNNNNAGTAC	oligo
220	AA**CTGCAG**TTACTTGTACAGCTCGTCCAT	*Pst*I
301	AA**GGATCC**GTTTGTACAATTCATCCATACCAT	BamHI
404	CACTGCAGCAAAATCCATCGTA	qPCR
405	GCCGCGCTGCCTCTCTA	qPCR
549	AAAGGATCCTGATTATTTCACTTTAAG	*Bam*HI
550	CCAGAACCGTTCATTTGCCCAGAGA	oligo
551	TCTCTGGGCAAATGAACGGTTCTGG	oligo
552	TGCTTCATAGAGAGTCGGTTATTTGTAC	oligo
553	GTACAAATAACCGACTCTCTATGAAGCA	oligo
554	AAATCTAGAATTGCATCGAAACTAAAT	*Xba*I
571	GACTCTAGACCATGGTTGTGTC	oligo
573	TAACAGGTTGGCTGATAAGTCCCC	oligo
574	GACGTCGACTCTAGACCATGGTTGTG	oligo
689	AA**GGATCC**ATGAATGAGAATCACAAAGAGC	*Bam*HI
690	AA**CTGCAG**TTATAACGCCTTCATATGCTGC	*Pst*I
750	GGAATCCTTATCAAGGAGTCTC	qPCR

751	CTGGTAGGGGTTAACG	qPCR
752	GGGCCAAAGTTTTACACCCCAAT	qPCR
753	CGGCAAATACATCAGCCAGAAAT	qPCR
754	CGAGCAGAATACATGCATCGGTTT	qPCR
755	GGCAACAGCAGCCTGAATCCC	qPCR
780	GACCGGGGACTTATCAGCC	oligo
781	GGCCAGCGAGCTAACGAGAC	oligo
1048	CGGTACCCGG**GGATCC**CATGCCGGAAACAATTATTAACAT	*Bam*HI
1049	CCAAGCTTGCATGC**GTCGAC**CTAAGCCCCCTTGCCCGG	*Sal*I
1054	GATC**GGATCC**ATGAATGAAAATCATAAAGAGC	*Bam*HI
1055	GATC**GAATTC**TCACGGTGTTCTTCAGTAGTCCA	*Eco*RI
1058	CGGTACCCGG**GGATCC**ATGATTTTAAAAACTATAAAAG	*Bam*HI
1059	CCAAGCTTGCATGC**CTGCAG**TTAGCATAGATTTATATCAGC	*Pst*I
1068	CTTGCATGC**CTGCAG**TCACTTGTCATCATCGTCCTTATAGTCACCTGATGACTTCTTGTGGC	*Pst*I
1069	CTTGCATGC**CTGCAG**TCACTTGTCATCATCGTCCTTATAGTCGCATAGATTTATATCAGCTTGAG	*Pst*I
1072	CTTGCATGC**CTGCAG**TCACTTGTCATCATCGTCCTTATAGTCGGATGAGGCATCCGTCACC	*Pst*I
1073	CGGTACCCGG**GGATCC**ATGCTCGAAATGTTAATGCAATG	*Bam*HI

1. Rao, C., H. Benhabib, and A.W. Ensminger, Phylogenetic reconstruction of the *Legionella pneumophila* Philadelphia-1 laboratory strains through comparative genomics. *PLoS One*, 2013. 8(5): p. e64129.
2. Berger, K.H. and R.R. Isberg, Two distinct defects in intracellular growth complemented by a single genetic locus in *Legionella pneumophila*. *Mol Microbiol*, 1993. 7(1): p. 7-19.
3. Zuckman, D.M., J.B. Hung, and C.R. Roy, Pore-forming activity is not sufficient for *Legionella pneumophila* phagosome trafficking and intracellular growth. *Mol Microbiol*, 1999. 32(5): p. 990-1001.
4. Coers, J., et al., Identification of Icm protein complexes that play distinct roles in the biogenesis of an organelle permissive for *Legionella pneumophila* intracellular growth. *Mol Microbiol*, 2000. 38(4): p. 719-36.
5. Kingry, L.C. and J.M. Petersen, Comparative review of *Francisella tularensis* and *Francisella novicida*. Front Cell Infect Microbiol, 2014. 4: p. 35.
6. Hanahan, D., Studies on transformation of *Escherichia coli* with plasmids. *J Mol Biol*, 1983. 166(4): p. 557-80.
7. Ninio, S., et al., The *Legionella* IcmS-IcmW protein complex is important for Dot/Icm-mediated protein translocation. *Mol Microbiol*, 2005. 55(3): p. 912-26.
8. Murata, T., et al., The *Legionella pneumophila* effector protein DrrA is a Rab1 guanine nucleotide-exchange factor. *Nat Cell Biol*, 2006. 8(9): p. 971-7.
9. Neild, A.L. and C.R. Roy, *Legionella* reveal dendritic cell functions that facilitate selection of antigens for MHC class II presentation. *Immunity*, 2003. 18(6): p. 813-23.
10. Nagai, H., et al., A C-terminal translocation signal required for Dot/Icm-dependent delivery of the *Legionella* RalF protein to host cells. *Proc Natl Acad Sci U S A*, 2005. 102(3): p. 826-31.
11. Andrews, H.L., J.P. Vogel, and R.R. Isberg, Identification of linked *Legionella pneumophila* genes essential for intracellular growth and evasion of the endocytic pathway. *Infect Immun*, 1998. 66(3): p. 950-8.
